# Identification of ALP+/CD73+ defining markers for enhanced osteogenic potential in human adipose-derived mesenchymal stromal cells by mass cytometry

**DOI:** 10.1186/s13287-020-02044-4

**Published:** 2021-01-06

**Authors:** Daisy D. Canepa, Elisa A. Casanova, Eirini Arvaniti, Vinko Tosevski, Sonja Märsmann, Benjamin Eggerschwiler, Sascha Halvachizadeh, Johanna Buschmann, André A. Barth, Jan A. Plock, Manfred Claassen, Hans-Christoph Pape, Paolo Cinelli

**Affiliations:** 1 Department of Trauma, University Hospital Zurich, Rämistrasse 100, 8091 Zurich, Switzerland; 2 Life Science Zurich Graduate School, University of Zurich, Winterthurerstrasse 190, 8057 Zurich, Switzerland; 3 Department of Biology, Institute of Molecular Systems Biology, ETH Zurich, Otto-Stern-Weg 3, 8093 Zurich, Switzerland; 4 Mass Cytometry Facility, University of Zurich, Winterthurerstrasse 190, 8057 Zurich, Switzerland; 5 Department of Plastic and Hand Surgery, University Hospital Zurich, Rämistrasse 100, 8091 Zurich, Switzerland

**Keywords:** Adipose-derived mesenchymal stromal cells, Stromal vascular fraction, Osteogenic potential, CyTOF, Multidimensional analysis, Cell subpopulation

## Abstract

**Background:**

The impressive progress in the field of stem cell research in the past decades has provided the ground for the development of cell-based therapy. Mesenchymal stromal cells obtained from adipose tissue (AD-MSCs) represent a viable source for the development of cell-based therapies. However, the heterogeneity and variable differentiation ability of AD-MSCs depend on the cellular composition and represent a strong limitation for their use in therapeutic applications. In order to fully understand the cellular composition of MSC preparations, it would be essential to analyze AD-MSCs at single-cell level.

**Method:**

Recent advances in single-cell technologies have opened the way for high-dimensional, high-throughput, and high-resolution measurements of biological systems. We made use of the cytometry by time-of-flight (CyTOF) technology to explore the cellular composition of 17 human AD-MSCs, interrogating 31 markers at single-cell level. Subcellular composition of the AD-MSCs was investigated in their naïve state as well as during osteogenic commitment, via unsupervised dimensionality reduction as well as supervised representation learning approaches.

**Result:**

This study showed a high heterogeneity and variability in the subcellular composition of AD-MSCs upon isolation and prolonged culture. Algorithm-guided identification of emerging subpopulations during osteogenic differentiation of AD-MSCs allowed the identification of an ALP+/CD73+ subpopulation of cells with enhanced osteogenic differentiation potential. We could demonstrate in vitro that the sorted ALP+/CD73+ subpopulation exhibited enhanced osteogenic potential and is moreover fundamental for osteogenic lineage commitment. We finally showed that this subpopulation was present in freshly isolated human adipose-derived stromal vascular fractions (SVFs) and that could ultimately be used for cell therapies.

**Conclusion:**

The data obtained reveal, at single-cell level, the heterogeneity of AD-MSCs from several donors and highlight how cellular composition impacts the osteogenic differentiation capacity. The marker combination (ALP/CD73) can not only be used to assess the differentiation potential of undifferentiated AD-MSC preparations, but also could be employed to prospectively enrich AD-MSCs from the stromal vascular fraction of human adipose tissue for therapeutic applications.

**Supplementary Information:**

The online version contains supplementary material available at 10.1186/s13287-020-02044-4.

## Introduction

Surgical interventions for bone repair are required for numerous reasons, such as trauma-resulting non-union fractures, or diseases including osteoporosis and osteonecrosis. Currently, autologous bone grafting is the most commonly used approach, but has a number of shortcomings such as the limited amount of harvested spongiosa and donor site pain [1]. Alternative approaches, including the use of synthetic bone substitutes, are not optimal because they lack the osteoinductive properties which are extremely important for healing large bone defects [2]. Cell therapies based on ex vivo expanded mesenchymal stromal stem cells (MSCs) in combination with appropriate scaffolds may be valuable alternatives to autologous bone grafting [3]. MSCs hold the ability to differentiate into osteoblasts and are available from a wide variety of tissue sources [4]. In particular, human fat tissue has been demonstrated to be a valuable source of MSCs—the so-called adipose-derived stromal cells (AD-MSCs) [3]. An additional advantage of using fat tissue is the relatively simple isolation procedure compared to autologous bone isolation [5]. We and others have shown that the combination of AD-MSCs in association with synthetic calcium phosphate bone substitutes may be a good alternative to autologous bone grafting [6–10]. Nevertheless, there are drawbacks linked to the use of MSCs for clinical therapy in humans. In contrast to other stem cell types (e.g., embryonic stem cells), the mechanisms that regulate self-renewal and lineage specification in MSCs are largely unexplored. In particular, MSC heterogeneity exists among donors, tissue sources, and within cell populations [11–14]. The knowledge regarding how different functional and differentiation attributes of MSCs are specified at the population level is insufficient. This poses significant obstacles in efforts to develop clinical manufacturing protocols that reproducibly generate functionally equivalent MSC populations [15, 16]. Currently, MSCs are defined by cell surface phenotypes, as well as their functional ability to differentiate into multiple cell lineages including osteoclast, chondrocyte, adipocyte, or skeletal myocyte lineages [17–19]. With respect to the clinical application of MSCs, much effort has been directed toward the identification of unique cell surface markers that could be used to purify cells from tissues to homogeneity.

In 2006, the International Society for Cell Therapy (ISCT) published the minimal criteria for defining MSCs [20]. These criteria comprise, besides plastic adherence and trilineage differentiation potential (osteogenic, chondrogenic, and adipogenic), the expression of CD105, CD73, and CD90, coincident with the lack of the hematopoietic markers CD45, CD34, CD14, CD19b, CD79a, and HLA-DR [20]. Additional markers have been identified over the years and are widely accepted for characterizing MSCs [21–31]. Even though all these markers were identified through functional experiments, in the sense that they correlate with the trilineage potential of the cells, it is not clear how their distribution and expression correlate with the observed differentiation capacity. Furthermore, questions remain open regarding whether MSCs express any unique surface epitopes, and more importantly, it is unknown whether the epitopes described to date have value in predicting MSC function.

In recent years, it was attempted to identify subpopulations of MSCs that show enhanced bone regenerative capability. Of note, most of these studies used a limited number of markers alone or in combinations, thus making comparison and reproducibility of the data difficult. It would be therefore essential to be able to analyze the expression of the identified markers in toto and at single-cell level in order to fully understand which subpopulations are undergoing osteogenic lineage commitment.

Recent advances in single-cell technologies have allowed multidimensional, high-throughput, and high-resolution measurements of biological systems. In this study, we applied cytometry by time-of-flight (CyTOF) to explore the cellular composition of 17 human AD-MSCs, interrogating 31 markers at single-cell level. The goal of this study was to investigate the subcellular composition of AD-MSCs in their naïve state as well as during osteogenic commitment via unsupervised dimensionality reduction [32], as well as by supervised representation learning approaches [33]. The data obtained reveal for the first time, in an unbiased way and at single-cell level, the heterogeneity of AD-MSCs from several donors and highlight the presence of subpopulations of cells with osteogenic lineage commitment properties. This information is of paramount importance considering the emerging need of MSCs for biomedical applications.

## Results

### Classification of osteogenic differentiation ability of 17 human AD-MSCs

**Fig. 1:**
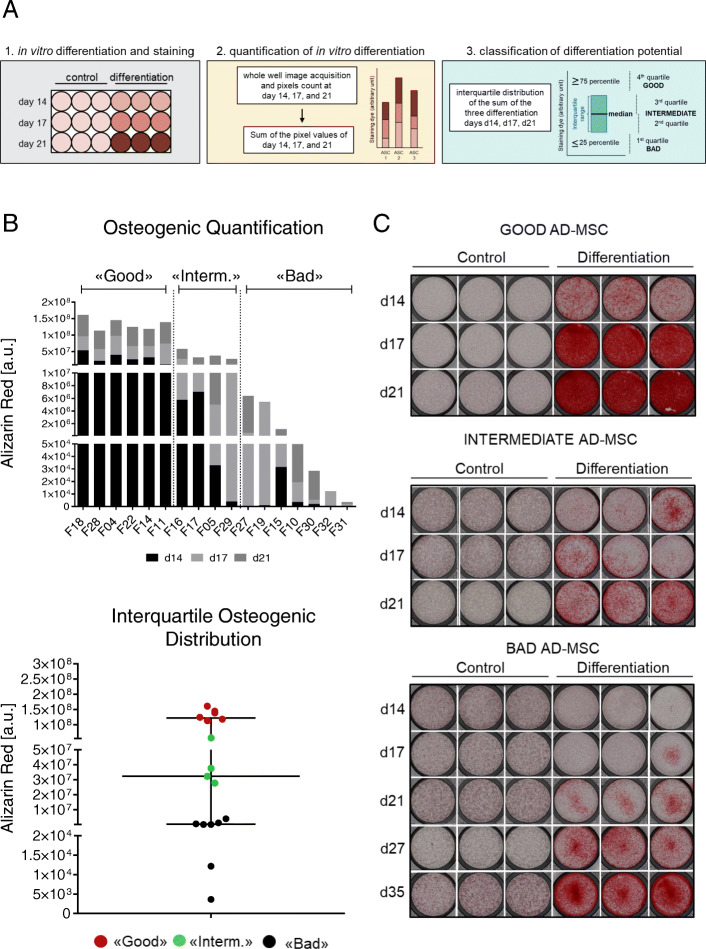
Classification of in vitro osteogenic differentiation potential of 17 AD-MSC lines. **a** Strategy used for the quantification of differentiation and AD-MSC classification: (1) Cells were differentiated in vitro into osteogenic lineage, and at three time points (days 14, 17, and 21), they were stained with Alizarin Red staining. (2) For each cell line, images of the whole well were acquired and pixels were counted and summed for the three time points (days 14, 17, and 21). (3) Interquartile distribution was applied, and it was decided that the 4th quartile was representing “good,” the 3rd and the 2nd quartile represented the “intermediate,” and the 1st quartile represented the “bad” differentiating lines. **b** Sum of the pixels acquired at the three time points (days 14, 17, and 21) for osteogenic differentiation of all 17 AD-MSC lines and interquartile categorization into “good,” “intermediate,” and “bad” AD-MSCs. **c** In vitro differentiation of one representing “good,” one “intermediate,” and one “bad” AD-MSC after 14, 17, and 21 days under osteogenic condition assessed by Alizarin Red staining. Depicted are triplicates of undifferentiated cells (control) and cells cultured under differentiation conditions

We have isolated 17 AD-MSCs from the stromal vascular fraction (SVF) of human fat tissue following standard protocols [34]. We further assessed the trilineage potential of the established cell lines by inducing differentiation toward osteogenic, chondrogenic, and adipogenic fate. Expression of lineage-specific markers during the differentiation process was monitored by RTQ-PCR (data not shown) and by classical staining assays (Alizarin Red, Alcian Blue, and Oil Red: Figs. 1 and S1A) at days 14, 17, and 21. Staining intensity was quantified using a highly standardized, automated digital image quantification approach [35]. This approach takes into consideration not only the amount of deposited dye in the whole cell culture dish but also the time needed for differentiation [35]. Shortly, for each cell line, the calculated pixels for each differentiation day (days 14, 17, and 21) were summed up to obtain one single value per line (Figs. 1a, b and S1A). Next, the lines were categorized into “good,” “intermediate,” and “bad” differentiating cells based on the interquartile range distribution. We categorized lines in the 1st quartile as “bad,” lines in the 2nd and 3rd quartile as “intermediate,” and lines in the 4th quartile as “good” (Figs. 1a, b and S1A). Cells from different donors clearly showed variable differentiation abilities (Figs. 1b, c and S1A). For example, in “good” osteogenic differentiating lines, calcium deposition was already detected at day 14 whereas “bad” lines did not show differentiation at day 21 but needed in average at least 30 days to fully differentiate (Fig. 1c). The “intermediate” AD-MSC lines showed Alizarin Red staining around day 17 and classified therefore between the “good” and the “bad” lines (Fig. 1c). A similar trend was also observed for chondrogenic and adipogenic differentiation (Figure S1A). Of interest, “good” lines for one lineage were not necessarily “good” for the other two lineages and the same was true for “bad” lines (Figure S1B). These data suggest either an impairment of the cells to differentiate or the existence of different subpopulations with varying differentiation potential.

### Single-cell, multidimensional analyses reveal high cellular heterogeneity in AD-MSCs

**Fig. 2:**
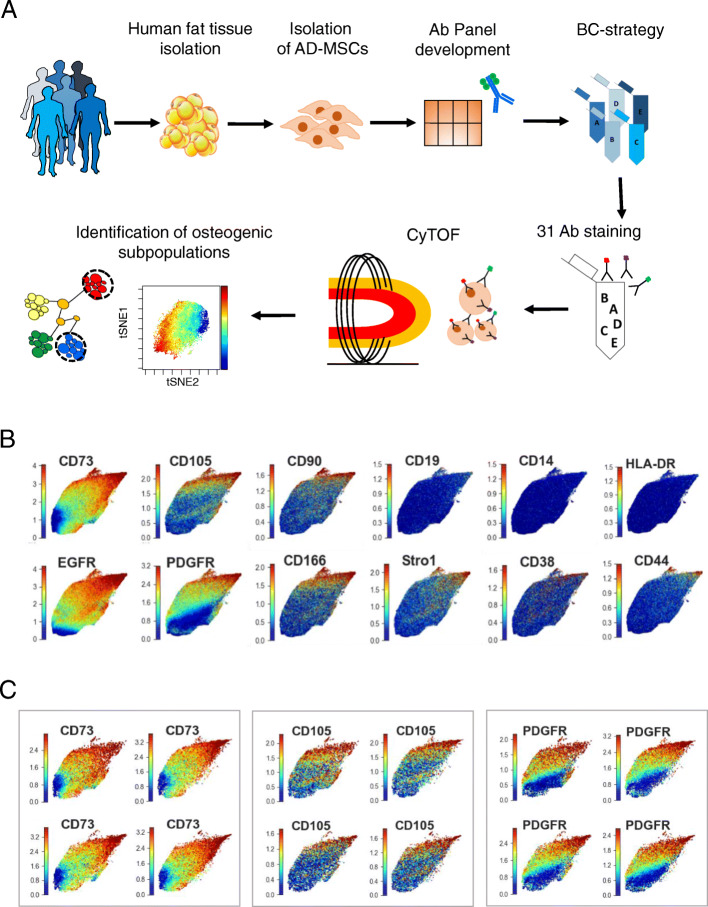
Mass cytometry analyses of human AD-MSCs reveal high heterogeneity. **a** Scheme of mass cytometry analysis on 17 human AD-MSCs from AD-MSC collection to the identification of osteogenic subpopulations. **b** UMAPs of selected markers in all 17 analyzed AD-MSC lines. **c** UMAPs of three selected markers (CD73, CD105, PDGFR) in 4 AD-MSC donors. Each dot represents one cell. Blue denotes minimal, green intermediate, and red high expression

In order to dissect the differences between the AD-MSCs obtained from different donors, we firstly performed single-cell analyses with mass cytometry at their naïve/undifferentiated state. CyTOF allows the simultaneous analysis at single-cell level of up to 50 different parameters using antibodies conjugated with metal isotopes [36, 37]. This technique combines flow cytometry and mass spectrometry and has already been used to unravel cellular heterogeneity in the context of cancer, immune diseases, or cellular differentiation [38–40] as well as for identifying subcellular markers for diseases [41]. However, this technology was never employed to characterize human AD-MSCs. We coupled the high dimensionality of mass cytometry with advanced cellular barcoding to simultaneously investigate 31 markers in 17 primary human AD-MSC lines to dissect at single-cell level their cellular composition (Fig. 2a).

Visualization of the distribution of the 31 markers in the AD-MSC lines with the dimensionality reduction method Uniform Manifold Approximation and Projection (UMAP) [32] highlighted the intra- and inter-donor heterogeneity (Figs. 2b, c and S2A). Interestingly, all 17 AD-MSC lines formed one compact cloud showing high degree of similarity among cells not only within the cell lines but also among donors (Figs. 2b, c and S2A). Despite the high degree of similarity among cells, the expression profiles of the investigated markers were not homogeneously distributed over the cloud but showed a gradient-like distribution all over the 17 AD-MSC lines. Interestingly, this was also the case for the widely accepted MSC markers CD73, CD105, and CD90 [20]. The expression of these key markers mostly co-localized in the same region of the cloud and was overlapping with the expression of other markers described in the literature to be critical for MSCs, such as EGFRα and PDGFRα (Figs. 2b and S2A). In agreement with the minimal criteria definition [20], the negative markers were indeed not expressed in the AD-MSC lines (Figs. 2b and S2A). Other markers such as CD146, NG2, CD271, and STRO-1 were expressed only by a relatively low number of cells and were heterogeneously distributed over the cloud (Figure S2A). We next generated UMAPs for each individual AD-MSC donor for all 31 markers. Although very small, each marker showed inter-donor variation regarding not only the amount of positive cells but also the expression intensity of the markers (Fig. 2c). These data clearly highlight in an unprecedented, multiparametric, and multidimensional way the heterogeneous composition of AD-MSC from several donors at single-cell level, suggesting the presence of specific subpopulations.

### Algorithm-guided identification of an emerging subpopulation during AD-MSCs’ osteogenic differentiation

**Fig. 3:**
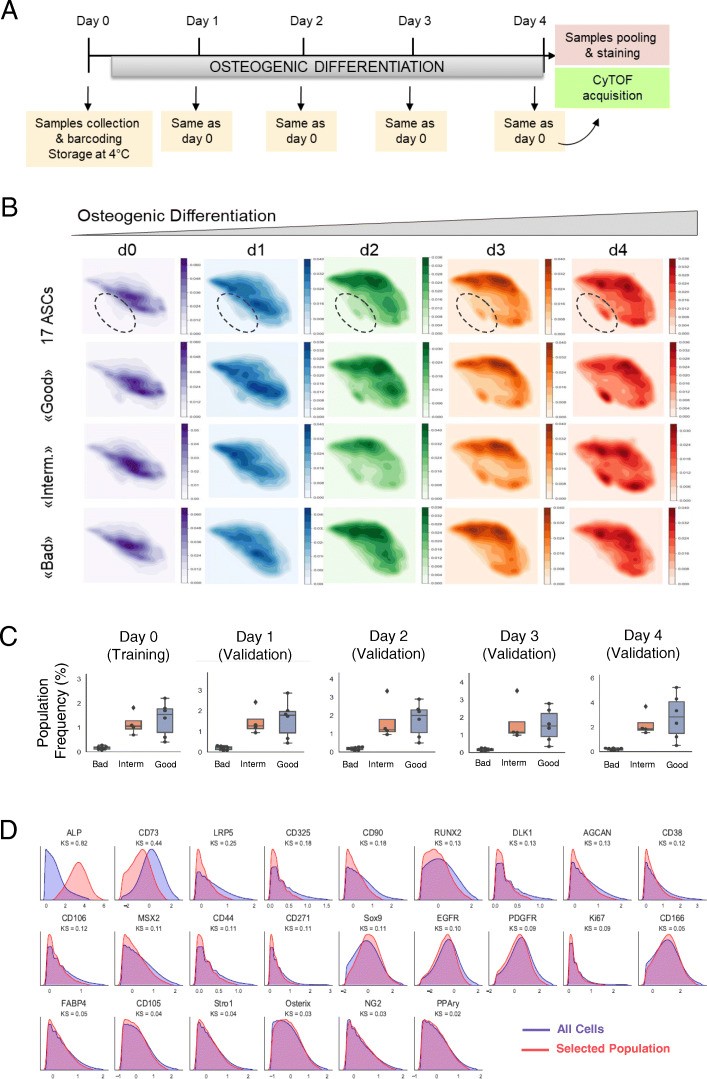
Identification of AD-MSC osteogenic subpopulation. **a** Sample collection and CyTOF approach scheme during 4 days of osteogenic differentiation (d0 = undifferentiated state, d1–d4 = differentiation). **b** Cell density plots on the UMAPs of the five analyzed days (d0, d1, d2, d3, d4) during osteogenic differentiation. Once the pool of all 17 AD-MSC lines is represented, once only the “good,” the “intermediate” (interm.), and the “bad” AD-MSC lines. Highlighted is the emerging population during osteogenic differentiation. Bright color indicates lower density, and dark color indicates higher cellular density. **c** Empirical distribution densities of all analyzed 31 marker abundances for the entire cell population (blue) and the cell subset selected by CellCNN (red). The identified subpopulation is characterized by alkaline phosphatase-positive (ALP+) and CD73low expressing cells. **d** Boxplots indicating the frequencies of the ALP+/CD73low subpopulation selected by CellCNN in all “good,” “intermediate” (interm.), and “bad” osteogenic differentiating lines during the five analyzed days. Error bars represent the mean of the percentage of positive cells present in “good” (*n* = 6), “intermediate” (*n* = 4), and “bad” (*n* = 7) AD-MSCs

We further wondered whether the variable differentiation ability of the “good” and “bad” AD-MSCs is due to the presence of specific subpopulations. Since it was previously shown that lineage specification occurs during the first 4 days of differentiation [42], we cultured all 17 AD-MSC lines under osteogenic condition and investigated at the single-cell level with CyTOF the population dynamics. Shortly, at five different time points, cells for each of the 17 AD-MSC lines were collected (day 0: undifferentiated cells, day 1–4: differentiation) and strategically barcoded (Table S2). At day 4, all samples were simultaneously stained and processed for CyTOF acquisition (Fig. 3a and Table S1).

Cell density plots on the UMAPs of the 17 AD-MSC lines during the initial 4 days of differentiation highlighted an emerging subpopulation, which was very small at day 0 and increased over the differentiation period (Fig. 3b). This subpopulation was clearly visible already at day 0 in the “good” lines whereas in the “bad” lines it was barely present even at day 4 (Fig. 3b). To further investigate whether it was possible to discriminate the differentiation potential of AD-MSC lines at their undifferentiated state, we applied the CellCNN algorithm [33] to the mass cytometry data obtained at day 0 (undifferentiated state). Presented with the task of comparing “good” versus “bad” cell lines, CellCNN detected a subpopulation characterized by high alkaline phosphatase (ALP+) expression and low expression of the MSC marker CD73 (CD73low) (Fig. 3c). This subpopulation was highly frequent in “good,” moderately present in the “intermediate,” and almost absent in the “bad” cell lines and was confirmed and validated on all later days of the differentiation process (Fig. 3d). Analysis of the percentages of cells positive for ALP and CD73 in each category always confirmed significant high frequency of ALP+ cells in the “good” lines, moderate frequency in the “intermediate” lines, and very low frequency of ALP+ cells in the “bad” lines over the four osteogenic days (Figure S3A). The percentage of CD73-positive cells was constant during the 4 differentiation days in the three categories, but significantly increased in the bad lines at day 2 and day 4 compared to “good” lines (Figure S3A).

We further investigated the correlation between ALP+ frequency (measured by CyTOF during the 5 days) and the ability to differentiate into osteocytes (based on the quantification of the staining at days 14, 17, and 21). As expected ALP+ always correlated with the osteogenic differentiation ability (Figure S3B) confirming once more that ALP+ expression correlates with osteogenic lineage commitment. In conclusion, our approach allowed the identification of an osteogenic subpopulation characterized by the markers ALP+/CD73low that hallmarked exclusively the “good” differentiating lines.

### ALP+/CD73+ cells possess enhanced osteogenic differentiation ability

**Fig. 4:**
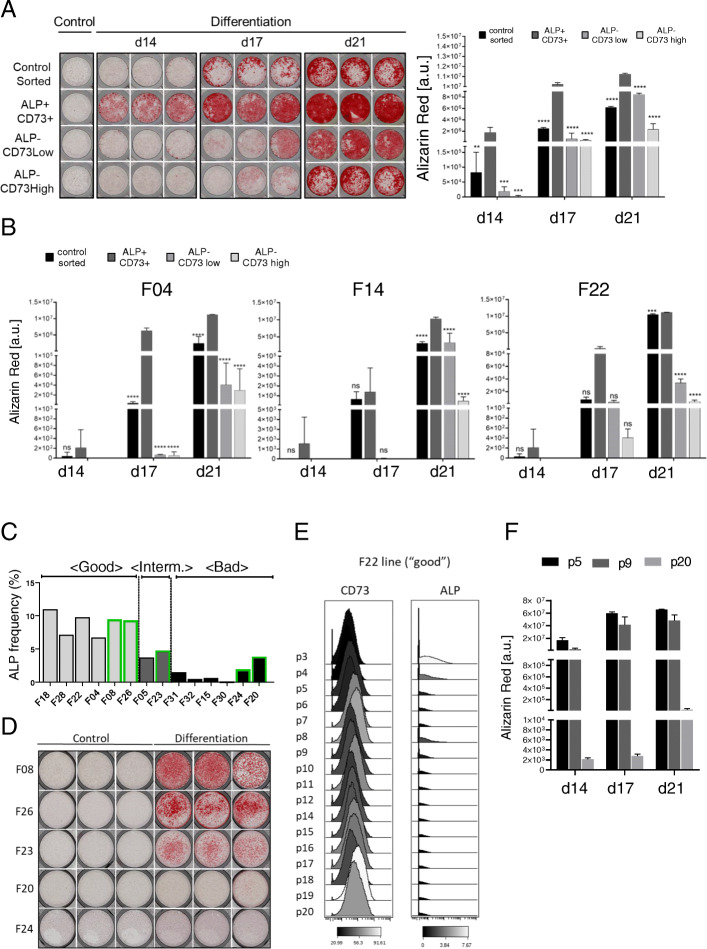
ALP+/CD73+ markers possess higher and predictive osteogenic potential. **a** Alizarin Red staining and quantification of F28 AD-MSC line sorted subpopulations (ALP+/CD73+, ALP−/CD73low, ALP−/CD73high) after 14, 17, and 21 days of osteogenic differentiation. Controls sorted are unstained cells, which were run through the FACS machine. Depicted is one triplicate of undifferentiated cells (control) and triplicates of cells cultured under osteogenic differentiation conditions (differentiation). **b** Quantification of Alizarin Red staining for F04, F14, and F22 AD-MSC lines for the same sorted subpopulations after 14, 17, and 21 days of osteogenic differentiation. Error bars indicate the triplicates of the staining and are presented as mean ± s.d. **c** Predicted categorization based on alkaline phosphatase (ALP) frequency in five new AD-MSC lines (green) and nine already characterized AD-MSC lines (reference) measured by CyTOF. **d** Alizarin Red staining at day 21 of the five new AD-MSC lines. Depicted are triplicates of undifferentiated cells (control) and cells cultured under osteogenic differentiation conditions (differentiation). **e** Histogram for the median intensity of expression of CD73 and ALP of F22 “good” AD-MSC line from passage 3 (p3) till passage 20 (p20). Black is the lowest intensity, and white represents the highest intensity. **f** Alizarin Red staining and quantification of F22 at passage p5, p9, and p20 after 14, 17, and 21 days of osteogenic differentiation. Error bars indicate the triplicates of the staining and are presented as mean ± s.d. For statistical analyses, the one-way ANOVA Dunnett’s multiple comparisons test was used to compare the ALP+/CD73+ population with the other sorted fractions within the same day: *p ≤ 0.05, **p ≤ 0.01, ***p ≤ 0.001, and ****p ≤ 0.0001. ns, not significant

In order to further characterize the identified subpopulation, we selected four AD-MSC lines (F28, F14, F04, and F22) and sorted three distinct cell subpopulations by FACS: ALP+/CD73+, ALP−/CD73low, and ALP−/CD73high. Although CellCNN analysis on CyTOF data revealed the presence of an ALP+/CD73low population, this phenotype was not clearly definable by FACS sorting. We could select ALP−/CD73high and ALP−/CD73low, but it was not possible to unambiguously distinguish between ALP+/CD73low and ALP+/CD73high cells. For this reason, we selected the double positive ALP+ and CD73+ (ALP+/CD73+) population for further experiments (Figure S4A). As a control, we used for each AD-MSC line unstained cells processed through the FACS. After sorting, the different subpopulations were directly plated for differentiation into the three lineages followed by lineage-specific staining at days 14, 17, and 21 and quantification according to Eggerschwiler et al. [35]. The sorted ALP+/CD73+ fraction showed enhanced osteogenic differentiation when compared to the other sorted populations (Figs. 4a, b and S4B).

### ALP+CD73+ marker combination is predictive for osteogenic potential in undifferentiated AD-MSC populations

We next assessed whether the marker ALP+ (which is also CD73+, see Figure S4A) could be used as a predictor for osteogenic differentiation potential of AD-MSCs in their undifferentiated state. For this purpose, we selected five new AD-MSC lines, which had never been characterized or used in previous experiments. Undifferentiated cells from the new lines, together with 9 already characterized lines (as reference cells), were subjected to CyTOF (Table S3). Quantification of the presence of ALP+ cells in the new lines allowed a predicted categorization based on the 9 AD-MSCs, into “good,” “intermediate,” and “bad” lines (Fig. 4c). We further compared the outcome from the CyTOF data with the differentiation ability observed in vitro (Fig. 4d). Alizarin Red quantification and the interquartile categorization of these lines confirmed two predicted “good” lines, one predicted “intermediate” line, and two predicted “bad” lines (Figs. 4c, d and S4C-D). Thus, we could confirm that the presence of the marker combination ALP+/CD73+ is sufficient to predict the osteogenic differentiation ability of a donor AD-MSC line in its undifferentiated state.

### ALP+CD73+ marker combination can be used for monitoring the osteogenic potential of undifferentiated AD-MSC populations after expansion in vitro

A major problem during in vitro expansion of MSCs (and also AD-MSCs) is that they show signs of aging and changes in the subcellular composition, which finally lead to a decrease of the differentiation potential over the passages [14]. To follow the dynamic of the cell composition over prolonged cell culture, we analyzed with our CyTOF antibody panel 3 “good” and 1 “intermediate” AD-MSC lines (F28, F14, F22, and F05) from passage 3 (p3) to passage 20 (p20) (Table S4). We could confirm that the median intensity of expression of CD73 was increasing whereas ALP was rapidly diminishing after prolonged culture, mirroring the situation observed in all “bad” lines at p10 (Figs. 4e and S4E).

We next differentiated the cells at p5, p9, and p20, and we observed a decrease in the differentiation capacity over the passages and these changes correlate with the expression of ALP and CD73 (Figs. 4e, f and S4F). In conclusion, we confirmed that ALP+/CD73+ expressing cells possess higher osteogenic differentiation potential and the marker combination of ALP and CD73 can be used to predict the osteogenic differentiation potential of cultured AD-MSCs.

### ALP+/CD73+ cells are present in the SVF of human fat tissue

**Fig. 5:**
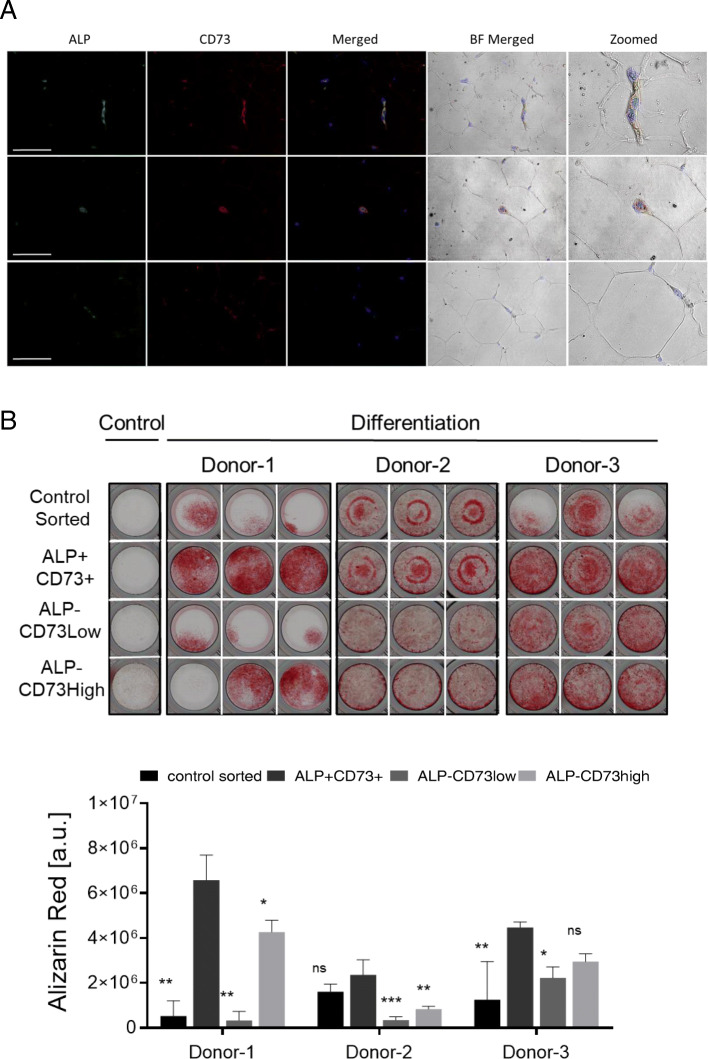
ALP+/CD73+ cells are present in human fat tissue and possess enhanced osteogenic potential. **a** Immunofluorescence of ALP and CD73 in human fat tissue. Scale 100 μm. BF, bright field. **b** Alizarin Red staining and quantification of 3 healthy donors’ SVF after 21 days of osteogenic differentiation. Depicted is one triplicate of undifferentiated cells (control) and triplicates of cells cultured under osteogenic differentiation conditions. Error bars indicate the triplicates of the staining and are presented as mean ± s.d. For statistical analyses, the one-way ANOVA Dunnett’s multiple comparisons test was used to compare the ALP+/CD73+ population with the other sorted fractions within the same day: *p ≤ 0.05, **p ≤ 0.01, and ***p ≤ 0.001. ns, not significant

To ultimately prove the clinical utility of the identified ALP and CD73 marker combination, we investigated whether ALP+/CD73+ cells were also present in freshly isolated human adipose stromal vascular fractions (SVFs), and if, upon isolation, they displayed similar properties as the ALP+/CD73+ cells present in AD-MSC lines. For this purpose, human adipose tissues were collected from 3 healthy donors, and the presence of ALP+/CD73+ cells was investigated. Immunohistochemical staining revealed the presence of ALP+/CD73+ located in fat tissue capillaries (Figs. 5a and S5A). SVFs from the same donors were further processed by FACS sorting, and the fractions (control: unstained cells sorted through FACS; CD45−/ALP+/CD73+, CD45−/ALP−/CD73low, CD45−/ALP−/CD73high) were plated for osteogenic differentiation (Figs. 5b and S5B). Quantification of osteogenic differentiation at d14, d17, and d21 confirmed higher osteogenic differentiation in the ALP+/CD73+ sorted cells compared to the other ones (Figs. 5b and S5C).

These data demonstrate that ALP+/CD73+ cells are present in freshly isolated human fat tissue and possess enhanced osteogenic potential, representing therefore interesting cells for therapeutic applications.

## Discussion

Even with the most effective protocols, different MSC preparations show strong variation in their differentiation performance. One possible explanation for this phenomenon is the high heterogenic cellular composition of MSCs, consisting of different cells harboring diverse lineage commitment ability [43]. The high donor-to-donor variability observed when MSCs are derived from the same tissue of origin may be due to different factors including donor health [11, 44], age, MSC availability, and/or self-renewal capacity [45–47]. However, variability can also be observed when BM-MSCs were autologously isolated over different periods of time or even when isolated bilaterally from the same donor [15], indicating that the cellular composition of MSCs plays an important role and is highly heterogeneous. A possibility to explain this heterogeneity is the variable composition of the tissues used for the establishment of MSC lines (e.g., amount of blood vessels). Dissecting this heterogeneity at single-cell level and identifying subpopulations of cells with specific differentiation attributes are urgently needed for developing clinical manufacturing protocols that reproducibly generate functionally equivalent MSC populations.

In this study, we have aimed at identifying specific AD-MSC subpopulations of cells with higher osteogenic differentiation potential. The novel approach used herein enabled the simultaneous visualization of 31 selected markers in 17 primary AD-MSC lines, thereby offering unprecedented observational dimensionality in a large sample set. This approach allowed circumvention of the classical bulk assays most frequently used for characterizing MSCs and their differentiation potential, which pool signals across entire cell populations, masking cell-to-cell variation. Unexpectedly, the dimensionality reduction algorithm UMAP revealed a high degree of cellular similarity, as observed from the compact clouds that all AD-MSC lines generated. This is in contrast to hematopoietic cells, for example, where UMAPs clearly separate the different cell subpopulations (Bendall et al. [37]). Nevertheless, despite the high degree of similarity among cells, the distribution of the markers within the clouds was highly heterogeneous, forming in some cases gradients (such as CD73, EGFR, PDGFR, SOX9) or small islands (ALP, CD166, STRO-1) (Figs. 2b and S2A). Furthermore, each marker showed inter-donor variation regarding not only the amount of positive cells but also the expression intensity of the markers (Fig. 2c).

Although ALP and CD73 have never been associated together with osteogenic potential, singularly they were previously correlated with osteogenic differentiation. CD73 was shown to regulate bone formation and remodeling in intramembranous bone repair [48]. In our study, we demonstrated that CD73 expression levels inversely correlate with the osteogenic differentiation ability of 17 human AD-MSC primary preparations (Figs. 4e, f and S4E-F). Tissue nonspecific ALP has been found in several tissues and cell types, such as activated B cells or pluripotent embryonic stem cells [49, 50], and it is an accepted osteoblast marker. CD73 and ALP are GPI (glycophosphatidylinositol)-anchored ectoenzymes with 5′-nucleotidase activity; thus, they share similar functions. CD73 and ALP regulate the extracellular breakdown of ATP to adenosine [51]. Released ATP serves as an autocrine or paracrine regulator of both osteoblast and osteoclast functions [52, 53], and hydrolyzation of pyrophosphate provides inorganic phosphate to promote mineralization. The extracellular nucleotide ATP can be one of the key mediators in bone metabolism, not only as a phosphate source, but also as a signaling molecule via P2 receptors. In fact, osteoblasts have been reported to release ATP into the extracellular environment constitutively followed by engagement of P2 receptors [54]. Most importantly, ALP+/CD73+ cells are also present and even more abundant in freshly isolated SVFs. The origin of these cells has to be better characterized, but it is reasonable to assume that these cells could be of pericytic origin. ALP is a known pericytic marker which was previously described as a marker for the prospective isolation of pericytes from different tissues [55, 56]. This is in agreement with our observation that ALP+/CD73+ cells are localized in the capillaries of fat tissue. In this sense, the difference observed between “good” and “bad” AD-MSC lines could be explained with differences in the amount of blood vessels in the isolated fat tissue. Our data suggest the existence of a balanced regulation of ALP and CD73 in human AD-MSCs, which is crucial for the determination of osteogenic lineage commitment.

In vitro selection after prolonged culture represents a major concern for the use of MSCs for therapeutic applications. Expansion on hard tissue culture surfaces may promote cellular divergence and/or reduction in potency [57, 58]. Additionally, the culture conditions used are very permissive when compared with the ones employed by other stem cell types, e.g., embryonic stem cells or induced pluripotent stem cells, where specific factors are necessary to maintain the self-renewal capacity of the stem cells [59–61]. Our data indicate that progressive loss of ALP+/CD73low cells during passaging precludes osteogenic differentiation and constantly monitoring ALP+/CD73low can be used as a quality control procedure to monitor AD-MSC expansion for bone regeneration purposes.

In conclusion, our study highlights that single-cell and multiparametric analysis identifies gradient expression and co-localization of markers which have not been previously observed. The combination of ALP+/CD73low markers can not only (1) discriminate between “good” and “bad” differentiating lines but can also be used for (2) prospective isolation of selected cells from SVF for bone tissue engineering and (3) to assess the differentiation potential of AD-MSC preparation in culture.

The use of MSCs in clinical medicine will likely continue to grow rapidly, yet it still is unclear how clinical manufacturing affects MSC biology, particularly regarding lineage specification. The development of assays allowing for the monitoring of the production process and assessment of cellular function are urgently needed. The approach chosen in this work might provide a basis for better understanding how different functional attributes of MSCs are specified at the population level, and can be used in the development of clinical manufacturing protocols that reproducibly generate functionally equivalent MSC populations.

## Material and methods

### Ethics statement

Adipose-derived stromal cells (AD-MSCs) were obtained from lipectomies and liposuctions (healthy donors, no diabetic donors) upon written informed consent of the donors, following the guidelines approved by the Kantonale Ethik Kommission (KEK) Zurich Swiss (KEK-ZH: StV 7-2009) and international ethical guidelines (ClinicalTrials.gov; Identifier: NCT01218945). The stromal vascular fraction (SVF) isolated from human fat tissue was obtained with the consent of the patient according to Swiss ethics (BASEC-Nr.: 2019-01504).

### Cells and cell culture

Twenty-two human adipose tissue samples (100–600 g) were obtained from lipectomies and liposuctions (healthy donors, no diabetic donors) [62]. AD-MSCs were isolated from fat tissue, with the consent of the donors according to Swiss (KEK-ZH: StV 7-2009) and international ethical guidelines (ClinicalTrials.gov; Identifier: NCT01218945) [62]. The extraction procedure was performed according to [34]*.* AD-MSCs were cultured in Dulbecco’s modified Eagle’s medium (DMEM) (PAN Biotech) supplemented with 10% of fetal bovine serum (FBS) (Biowest), 1% of antibiotics (100× penicillin, 100× streptomycin) (Biowest), and 1% l-glutamine 200 mM (Sigma) (called AD-MSC medium). Medium was changed every 3 days, and cells were passaged with 1× Trypsin-EDTA (Life Technologies) for 5 min at 37 °C when cells were about 80% confluent. Cells were incubated at 37 °C in an atmosphere with 95% humidity and 5% CO_2_.

### In vitro differentiation of human AD-MSCs

For osteogenic differentiation, AD-MSCs were seeded at a density of 1.6 × 10^4^ cells/cm^2^ in Nunc™ 24-well plates (Thermo Fisher Scientific) or at a density of 1 × 10^4^ in 96-well plates (TPP). For adipogenic differentiation, cells were cultured at a density of 1.6 × 10^4^ cells/cm^2^ in Nunc™ 24-well plates (Thermo Fisher Scientific). Differentiation was started 24 h after seeding with StemPro® Osteogenesis Kit or StemPro® Adipogenesis Kit (Gibco/Life Technologies). For chondrogenic differentiation, cells were cultured at a density of 5 × 10^3^ cells/cm^2^ in a Nunc™ 24-well plate (Thermo Fisher Scientific) and differentiation was induced at the 4th day of culture using the StemPro® Chondrogenesis Kit (Gibco/Life Technologies). All media were changed every 4 days.

### Assessment and classification of trilineage differentiation potential

Differentiation assessment via specific staining was performed for all three differentiation lineages after 14, 17, and 21 days of differentiation. For Alizarin Red S (Sigma) staining, cells were washed with PBS and fixed with 4% (v/v) formaldehyde (Sigma) for 30 min at RT. Upon washing twice with ddH_2_0, Alizarin Red S solution (0.7 g Alizarin Red S diluted in 50 ml ddH_2_O at pH = 4.2) was added for 20 min at RT. Afterwards, cells were washed four times with ddH_2_O, dried, and stored in the dark until image acquisition. For Oil Red O (Sigma) staining, cells were washed once with PBS and fixed with 10% (v/v) formaldehyde (Roth) for 1 h at RT. Afterwards, cells were washed twice with ddH_2_O, rinsed twice with 60% (v/v) 2-propanole (Sigma) in ddH_2_0, and dried. Oil Red O working solution (0.15 g Oil Red O in 50 ml 60% (v/v) 2-propanole in ddH_2_O) was added for 10 min at RT. After four ddH_2_O washing steps, cells were dried and images were directly taken. For Alcian Blue 8GX (Sigma) staining, cells were washed with PBS and then fixed with 4% (v/v) formaldehyde (Sigma) for 20 min at RT. Afterwards, cells were washed twice with ddH_2_O and incubated for 3 min with 3% (v/v) acetic acid (Merck Millipore) in ddH_2_0. Alcian Blue solution (0.1 g Alcian Blue 8GX in 100 ml of 3% acetic acid in ddH_2_0 at pH = 2.5) was given for 1 h at RT. Cells were washed four times with ddH_2_O, dried, and stored in the dark until image acquisition. Images of the entire wells at days 14, 17, and 21 of differentiation were acquired with Cytation 5 imaging reader (BioTek). Quantification of differentiation was performed according to [35], and subsequent classification of AD-MSC into “good,” “bad,” and “intermediate” differentiating lines was performed applying the interquartile range distribution. We defined cell lines present in the 4th quartile as “good,” lines present in the 2nd and 3rd as “intermediate,” and lines in the 1st quartile as “bad.”

### Isolation of the stromal vascular fraction

Stromal vascular fraction (SVF) was isolated from human fat tissue with the consent of the patient according to Swiss ethics (BASEC-Nr.: 2019-01504) and according to [34]. Briefly, lipectomies were cut in small pieces and extensively washed with PBS. Enzymatic digestion was performed with 0.075% collagenase I (Gibco) at 37 °C for 45 min in a rotating disk. The reaction was neutralized with AD-MSC medium and centrifuged at 850*g* for 10 min. For lysis of the red blood cells, the pellet was incubated for 10 min at RT in 160 mM NH_4_Cl and then extensively washed with PBS. The SVF was then filtered through a 100-μm filter nylon mesh and was either directly processed for FACS sorting followed by osteogenic differentiation, or frozen in AD-MSC medium supplemented with 10% DMSO (Sigma).

### Fluorescence activating cell sorting (FACS)

AD-MSC lines were washed with PBS and stained with ALP-APC (R&D) (1/50) and CD73-FITC (Biolegend) (1/160) for 25 min at 4 °C. Upon washing, the cell fractions (controls sorted, ALP+/CD73+, ALP−/CD73high, ALP−/CD73low) were sorted with a FACS BD Aria III 5L and seeded in Nunc™ 96-well plates (TPP) at a density of 1.2 × 10^4^ cells/cm^2^ for osteogenic differentiation. Controls sorted were unstained cells processed through the FACS and collected without sorting specific subpopulations. Differentiation was induced 24 h after seeding. Freshly isolated SVFs were washed with PBS and stained with ALP-APC (R&D) (1/50), CD73-FITC (Biolegend) (1/160), and CD45-PE (Biolegend) (1/160) for 25 min at 4 °C. SVF fractions (controls sorted, CD45−/ALP+/CD73+, CD45−/ALP−/CD73high, CD45−/ALP−/CD73low) were sorted with FACS BD Aria III 5L and plated in vitro at a density of 1 × 10^4^ in 96-well plates (TPP) for osteogenic differentiation. All media were changed every 4 days.

### Immunohistochemistry and immunofluorescence

Paraffin-embedded samples of human fat tissue were selected for immunohistochemical and immunofluorescence analysis. Samples were deparaffinized with xylene and rehydrated by an increasing ethanol gradient for hematoxylin and eosin (H&E) staining. Target retrieval was performed using the PT Link (DAKO) at pH solution 9.0 (DAKO). Immunohistochemistry staining was performed using a Dako Autostainer Link 48. Primary antibodies used were as follows: rabbit monoclonal ALP (Abcam, 1/200), mouse monoclonal CD73 (Abcam, 1/200), mouse monoclonal CD31 (DAKO, 1/200), and the appropriate EnVision HRP secondary antibody (EnVision HRP rabbit or mouse, DAKO, 1/500) according to the manufacturer’s instruction. Immunofluorescence was performed using a Dako Autostainer Link 48 with the following antibodies: rabbit monoclonal ALP (Abcam, 1/200), mouse monoclonal CD73 (Abcam, 1/200), Alexa Fluor 488 goat anti-rabbit IgG (Thermo Fisher, 1/200), and Alexa Fluor 546 goat anti-mouse IgG (Thermo Fisher, 1/200) according to the manufacturer’s instruction. Sections were visualized with LEICA DM6600 with a × 20 magnifying objective lens.

### Mass cytometry antibody panel and staining procedures

The antibody panel consisted of 31 monoclonal anti-human metal-conjugated antibodies, which included cell surface, cytoplasmic, and transcription targets (Table S1). When possible, already metal-conjugated antibodies were purchased from Fluidigm; otherwise, antibodies were conjugated in-house with isotopically pure lanthanide metals according to the commercially available MaxPar Antibody Labelling Kit (Fluidigm). Labeled antibodies were stored at 4 °C in antibody stabilizer solution (Candor Bioscience). Titration of each antibody was performed on a one-to-one mix of cells consisting of PBMCs (peripheral blood mononuclear cells), HEK (human embryonic kidney cells 293), Hela (cervical cancer cells), Jurkat (human T lymphocyte cells), Saos2 (sarcoma cells), Nalm6 (B cell precursor leukemia cells), SHSY5S (neuroblastoma cells), and human AD-MSCs. These different cell lines, which we called MIX, were chosen in order to have for each marker a positive and a negative control cell type. Sample staining was performed as described in the MaxPar Cell Surface, MaxPar Cytoplasmic/Secreted Antigen, and MaxPar Nuclear Target protocols (Fluidigm) with minor changes. Briefly, cells were first subjected to cell surface antibody staining, followed by cytoplasm staining, and nuclear staining. For the cytoplasmic and intranuclear staining, cell fixation steps were shortened to 10 min. Cells were then resuspended in 4% paraformaldehyde (Electron Microscopy Sciences) and stored at 4 °C until acquisition. In the day of CyTOF acquisition, cells were washed with MaxPar Fix and Perm Buffer (Fluidigm) containing Cell-ID Intercalator-IR (Fluidigm) and incubated at RT for 1 h. Cells were washed with ddH_2_O and then diluted in ddH_2_O with 10% EQ Calibration Beads (Fluidigm) at 1 million cells/ml before acquisition with CyTOF 2 mass cytometer (Fluidigm).

### Mass-tag cellular barcoding

For all CyTOF experiments, the Cell-ID 20-Plex Pd Barcoding Kit (Fluidigm) was used following the manufactural instructions. In short, 1 million cells per condition and per line were washed with PBS and then incubated with Cell-ID Cisplatin (Fluidigm) for 10 min at RT. Afterwards, cells were fixed with MaxPar Fix Buffer (Fluidigm) for 10 min at RT, washed with MaxPar Barcode Perm Buffer (Fluidigm), and incubated with the appropriate barcode for 30 min at RT. Finally, cells were washed with Cell Staining Buffer (Fluidigm) and combined depending on the CyTOF experiment in one or more tubes before antibody staining. Depending on the planned CyTOF experiment, a specific barcoding strategy was developed in order to minimize technical bias and highlight biological differences.

### Barcoding strategies for the osteogenic differentiation experiments

For this differentiation experiment, we had a total of 102 samples. Thus, having only 20 different barcodes available, we distributed the barcoded samples into 6 tubes (Tables S2). In each tube when possible, there was one “good,” one “bad,” and one “intermediate” line for all the collected time points. The 17 AD-MSC lines cultured under osteogenic condition were collected during the first 5 days (day 0, day 1, day 2, day 3, day 4) of differentiation. At each day, the samples were barcoded, pooled into the appropriate tube, and stored at 4 °C until day 4. At day 4, a unique antibody master mix was prepared and distributed into the six tubes. In order to monitor tube-to-tube variations, we added to each of the six tubes twice the MIX (PBMCs, HEK, Hela, Jurkat, Saos2, Nalm6m, SHSY5S, AD-MSCs) for a total of 102 samples (Tables S2). Stability of the barcoded samples stored at 4 °C during the four collection days was extensively proved in preliminary tests (data not shown).

### Barcoding strategy for prediction of differentiation potential in five new AD-MSC lines

Five not yet characterized AD-MSC lines (new AD-MSCs) together with 9 already characterized AD-MSC lines (reference) were collected in their undifferentiated state (day 0). Next, together with one MIX, they were all barcoded according to the barcode plan (Table S3) and pooled into one single tube for antibody staining and CyTOF acquisition as described above.

### Barcoding strategy for the passage experiment

AD-MSCs F28, F22, F5, and F14 were cultured in AD-MSC medium in Nunc™ 6-cm plates (Thermo Fisher Scientific) and passaged when 90% confluence was reached. This was repeated from passage 3 (p3) to passage 20 (p20). At each passage, part of the cells was frozen in AD-MSC medium supplemented with 10% DMSO (Sigma). All AD-MSC lines from p3 to p20 were thawed the same day and barcoded according to the barcode plan (Table S4). All barcoded passages from the same cell line were pooled into one tube. Each tube contained also twice a MIX as a control. Cells were stained with the antibody panel following the protocols mentioned above and then processed in CyTOF2 (Fluidigm).

### Mass cytometry data analysis

Mass cytometry data.fcs files collected from each set of samples were normalized using the executable MATLAB version of the Normalizer tool [63] and concatenated using the .fcs concatenation tool from Cytobank. Individual samples were debarcoded using the executable MATLAB version of the single-cell debarcoder tool [64].

### Statistical analyses

Quantification of the staining of the triplicates of undifferentiated cells (control) and cells cultured with differentiation medium (differentiation) is presented as mean ± s.d. Quantification of the triplicates of the staining of the FACS sorted subpopulations is presented as mean ± s.d. For statistical analyses, the one-way ANOVA Dunnett’s multiple comparisons test was used to compare the ALP+/CD73+ population with the other sorted fractions within the same day as well as for comparing the percentage of ALP+, CD73+, and CD271+ cells in the “good” category for each day with the same day of the “intermediate” and “bad” ones. *p ≤ 0.05, **p ≤ 0.01, ***p ≤ 0.001, and **** *p* ≤ 0.0001. Pearson’s correlation was used to determine the correlation between the ALP frequency measured by CyTOF at days 0, 1, 2, 3, and 4 with the staining intensity measured at days 14, 17, and 21 for the osteogenic differentiation lineage.

### CellCNN analysis

#### Data pre-processing

Mass cytometry measurements were transformed using the inverse hyperbolic sine (arcsinh) function with a cofactor of 5 and subsequently median-centered on a per-marker basis.

#### Model training

CellCNN was trained with the objective to classify “good” versus “bad” AD-MSC lines from their corresponding mass cytometry measurements at day 0 (undifferentiated state). Training examples (multi-cell inputs) comprised 2000 cells, sampled uniformly at random from the original mass cytometry samples. In total, we sampled 1000 training examples per class (“good” or “bad” cell lines). For the top-*k* pooling layer, we considered values of *k* such that the ratio of *k* over the multi-cell input size would be one of [0.5%, 1%, 3%, 5%]. The remaining CellCNN parameters were set to their default values.

#### Defining the selected cell subpopulation

The default CellCNN filter interpretation analysis was performed to define and characterize the selected cell subpopulation. Initially, learned filters were clustered and a single representative filter was retained from each cluster. As a second step, a score was derived for each representative filter, measuring how well this filter alone can classify the validation samples. Only one representative filter achieved a positive score, and this filter was used to define the selected cell subpopulation (i.e., cells with positive score with respect to that filter) in individual mass cytometry samples at d0, d1, d2, d3, and d4.

### Data availability

Mass cytometry data that support the findings of this study are available on request from the corresponding author [P.C.].

## Supplementary Information

**Additional file 1: Figure S1.** In vitro chondrogenic and adipogenic categorization of 17 AD-MSCs A) Sum of the pixels acquired at the three time points (day 14, 17, 21) for chondrogenic (left) and adipogenic (right) differentiation of all 17 AD-MSC lines and interquartile categorization into «good», «intermediate», and «bad» AD-MSCs. C) Summary of the categorization of all 17 AD-MSCs for the three differentiation lineages (osteogenic, chondrogenic, and adipogenic). interm. = intermediate. **Figure S2.** UMAP analyses in the 17 human AD-MSC lines. A) UMAP projections of all 31 markers in 17 AD-MSC lines. Each dot represents one cell. Blue denotes minimal, green intermediate, and red high expression. **Figure S3.** Analyses of the osteogenic subpopulation. A) Means of the percentage of alkaline phosphatase (ALP) positive cells and CD73 positive cells in the three AD-MSC categories during the five analyzed days of osteogenic differentiation (d0, d1, d2, d3, d4). Error bars represent the mean ± s.d. of the percentage of positive cells present in «good» (*n* = 6), «intermediate» (*n* = 4),and «bad» (*n* = 7) AD-MSCs. B) Pearson correlations of the ALP frequency measured by CyTOF at day 0, 1, 2, 3, 4 with the staining intensities measured at day 14, 17, and 21 for osteogenic differentiation. Red dots represent «good», green «intermediate» (interm.), and black «bad» differentiating lines. Error bars indicate the triplicates of the staining and are presented as mean ± s.d. For statistical analyses, the one-way ANOVA Dunnett’s multiple comparisons test was used to compare each day of the “good” AD-MSCs with the same day of “intermediate” and “bad” categories: * *p*≤ 0.05, ** *p*≤ 0.01, *** *p* ≤ 0.001, and **** *p* ≤ 0.0001. ns=not significant. **Figure S4.** ALP+/CD73+ Sorting analysis and prediction of osteogenic differentiation potential. A) Gating strategy for FACS sorting for the following subpopulations: ALP+/CD73+, ALP-/CD73low, and ALP-/CD73high. B) Alizarin Red staining and quantification of the sorted subpopulations in four AD- MSC lines (F04, F14, F22, F28) after 14, 17, and 21 days. Control sorted are unstained cells, which were run through the FACS sorting machine. Depicted are triplicates of undifferentiated cells (control) and cells cultured with the differentiation medium (differentiation). Error bars indicate the triplicates of the staining and are presented as mean ± s.d. C) Categorization of the new AD-MSC lines (depicted in green) together with all the 17 already analyzed lines, based on Alizarin Red quantification after 14, 17, and 21 days of osteogenic differentiation and interquartile distribution of the five new AD-MSCs (depicted in violet). D) Alizarin Red staining and quantification of five new AD-MSCs: two «good» (F08, F26), one «intermediate» (F23), and two «bad» (F20, F24). Depicted are triplicates of undifferentiated cells (control) and cells cultured under osteogenic differentiation conditions (differentiation). Error bars indicate triplicates of the staining and are presented as mean ± s.d. E) Histograms of median intensities of expression of selected markers (CD73 and ALP) in F05, F14, F22 and F28 AD-MSC lines from passage 3 (p3) till passage 20 (p20). Black is the lowest intensity and white represents the highest intensity. F) Alizarin Red staining and quantification of F22 at passage p5, p9, and p20 after 14, 17, and 21 days of osteogenic differentiation. Depicted are triplicates of undifferentiated cells (control) and cells cultured under osteogenic differentiation medium (differentiation). Error bars indicates the triplicates of the staining and are presented as mean ± s.d. **Figure S5.** ALP+/CD73+ cells are present in the human fat tissue and stromal vascular fraction A) Hematoxylin/Eosin (H&E) and immunohistochemistry staining of human fat tissue for ALP, CD73, and CD31. Scale 100 μm. B) Gating strategy for sorting the selected subpopulations (CD45- /ALP+/CD73+, CD45-/ALP-/CD73low, CD45-/ALP-/CD73high) in the SVFs. C) Alizarin Red staining and pixel quantification of sorted SVF fractions (CD45-/ALP+/CD73+, CD45-/ALP-/CD73low, CD45-/ALP- /CD73high) after 21 days of osteogenic differentiation in vitro. Control sorted are unstained SVFs, which were run through the FACS sorting machine. Depicted are triplicates of undifferentiated cells (control) and cells cultured with osteogenic differentiation medium (differentiation). Error bars indicate the triplicates of the staining and are presented as mean ± s.d. **Table S1**. Mass cytometry antibody panel. **Table S2**. Osteogenic differentiation barcoding schema. **Table S3**. Barcoding plan prediction experiment. **Table S4**: Barcoding plan for the passage experiment.
