# Supplementary material for: Identification of ALP+/CD73+ defining markers for enhanced osteogenic potential in human adipose-derived mesenchymal stromal cells by mass cytometry
Source: Stem Cell Res Ther. 2021 Jan 6;12:7. doi: 10.1186/s13287-020-02044-4 (PMC7789251; doi:10.1186/s13287-020-02044-4)
Supplement: Supplementary file 1 — Additional file 1: Figure S1. In vitro chondrogenic and adipogenic categorization of 17 AD-MSCs A) Sum of the pixels acquired at the three time points (day 14, 17, 21) for chondrogenic (left) and adipogenic (right) differentiation of all 17 AD-MSC lines and interquartile categorization into «good», «intermediate», and «bad» AD-MSCs. C) Summary of the categorization of all 17 AD-MSCs for the three differentiation lineages (osteogenic, chondrogenic, and adipogenic). interm. = intermediate. Figure S2. UMAP analyses in the 17 human AD-MSC lines. A) UMAP projections of all 31 markers in 17 AD-MSC lines. Each dot represents one cell. Blue denotes minimal, green intermediate, and red high expression. Figure S3. Analyses of the osteogenic subpopulation. A) Means of the percentage of alkaline phosphatase (ALP) positive cells and CD73 positive cells in the three AD-MSC categories during the five analyzed days of osteogenic differentiation (d0, d1, d2, d3, d4). Error bars represent the mean ± s.d. of the percentage of positive cells present in «good» (n = 6), «intermediate» (n = 4),and «bad» (n = 7) AD-MSCs. B) Pearson correlations of the ALP frequency measured by CyTOF at day 0, 1, 2, 3, 4 with the staining intensities measured at day 14, 17, and 21 for osteogenic differentiation. Red dots represent «good», green «intermediate» (interm.), and black «bad» differentiating lines. Error bars indicate the triplicates of the staining and are presented as mean ± s.d. For statistical analyses, the one-way ANOVA Dunnett’s multiple comparisons test was used to compare each day of the “good” AD-MSCs with the same day of “intermediate” and “bad” categories: * p≤ 0.05, ** p≤ 0.01, *** p ≤ 0.001, and **** p ≤ 0.0001. ns=not significant. Figure S4. ALP+/CD73+ Sorting analysis and prediction of osteogenic differentiation potential. A) Gating strategy for FACS sorting for the following subpopulations: ALP+/CD73+, ALP-/CD73low, and ALP-/CD73high. B) Alizarin Red staining and quantification of [file 13287_2020_2044_MOESM1_ESM.pdf]

Figure S1

A

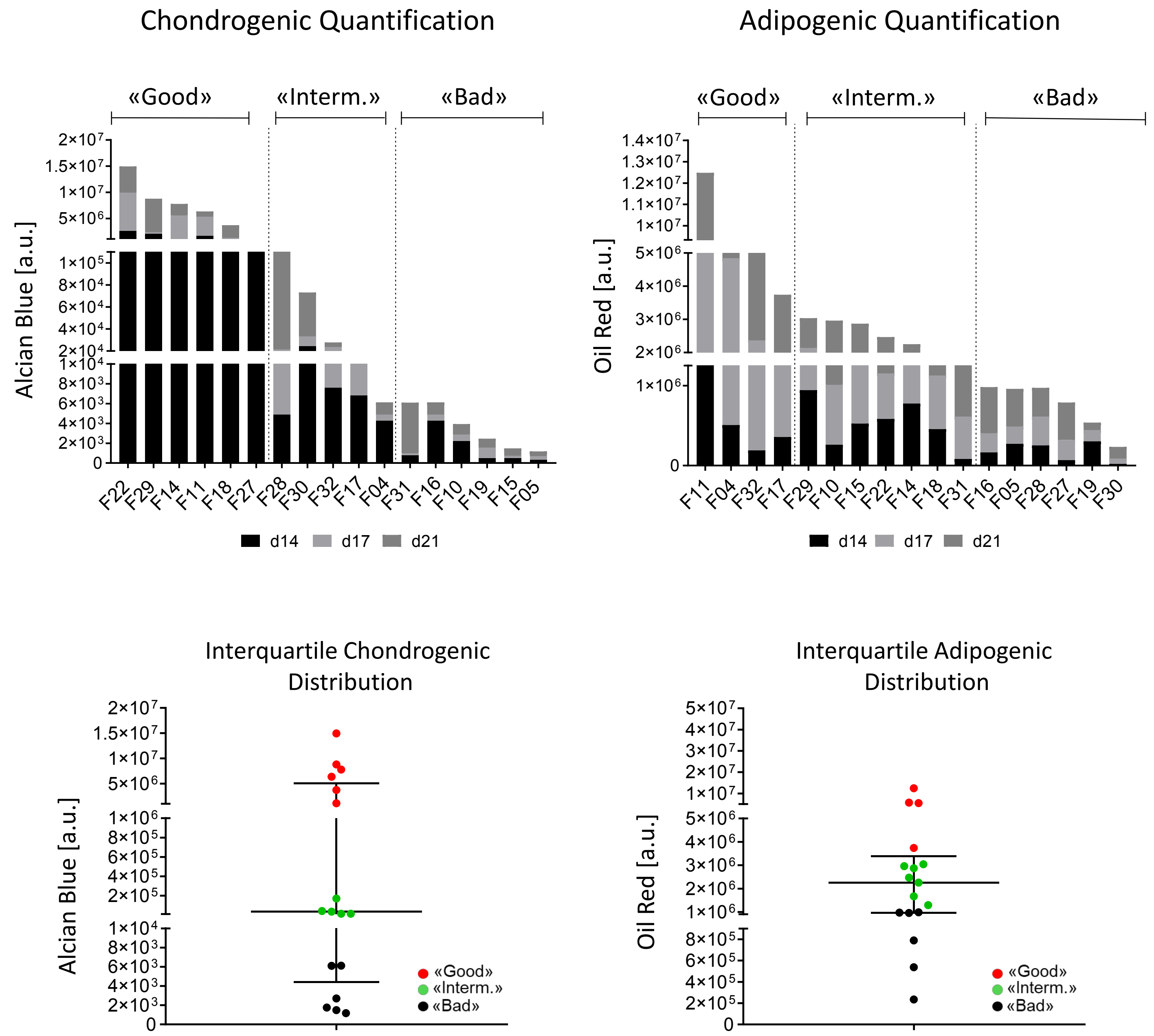

B

LINEAGE	«Good» ASCs	«Interm.» ASCs	«Bad» ASCs
**Osteogenic**	F18, F28, F04, F22, F14, F11	F16, F17, F05, F29	F27, F19, F15, F10, F30, F32, F31
**Adipogenic**	F11, F04, F32, F17	F10, F29, F15, F22, F14, F18, F31	F16, F05, F28, F27, F19, F30
**Chondrogenic**	F22, F29, F14, F11, F18, F27	F28, F30, F32, F17, F04	F31, F16, F10, F19, F15, F05

Figure S2

A

All AD-MSCs

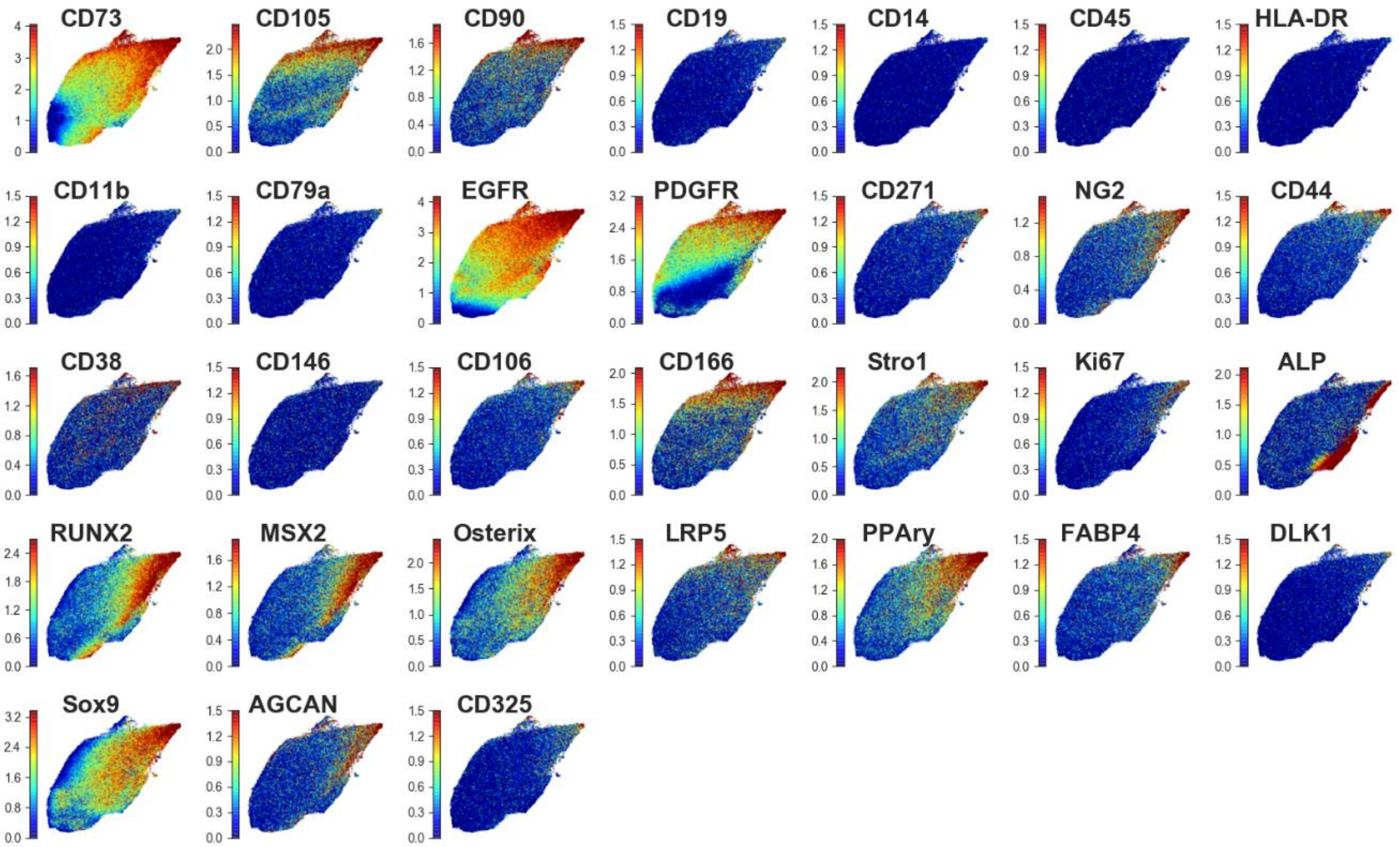

Figure S3

A

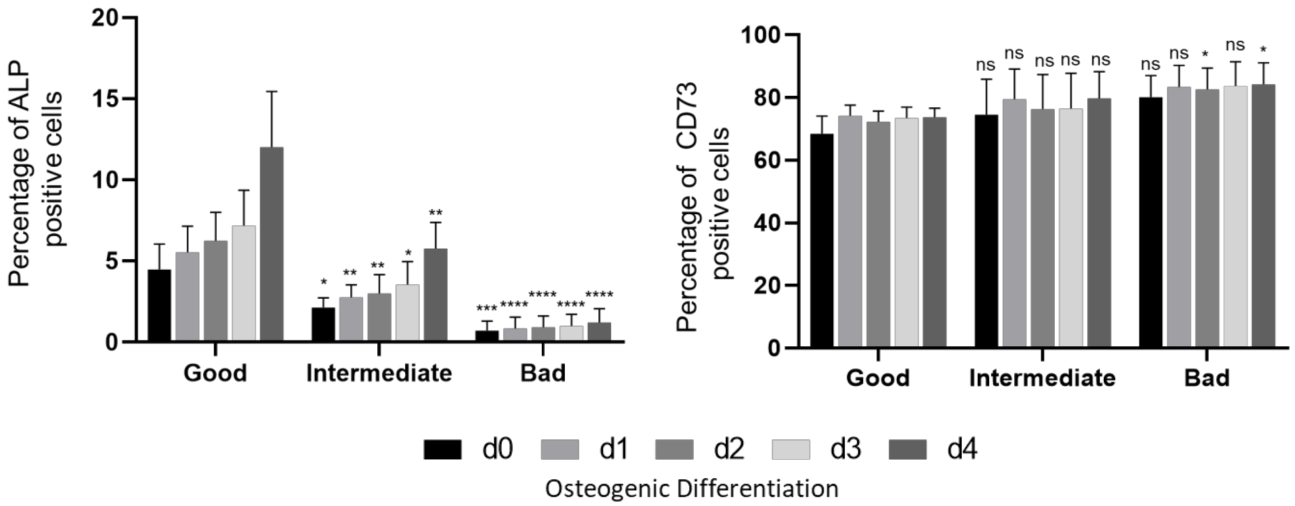

Percentage of ALP positive cells
Good
Intermediate
Bad
Percentage of CD73 positive cells
d0
d1
d2
d3
d4
Osteogenic Differentiation

B

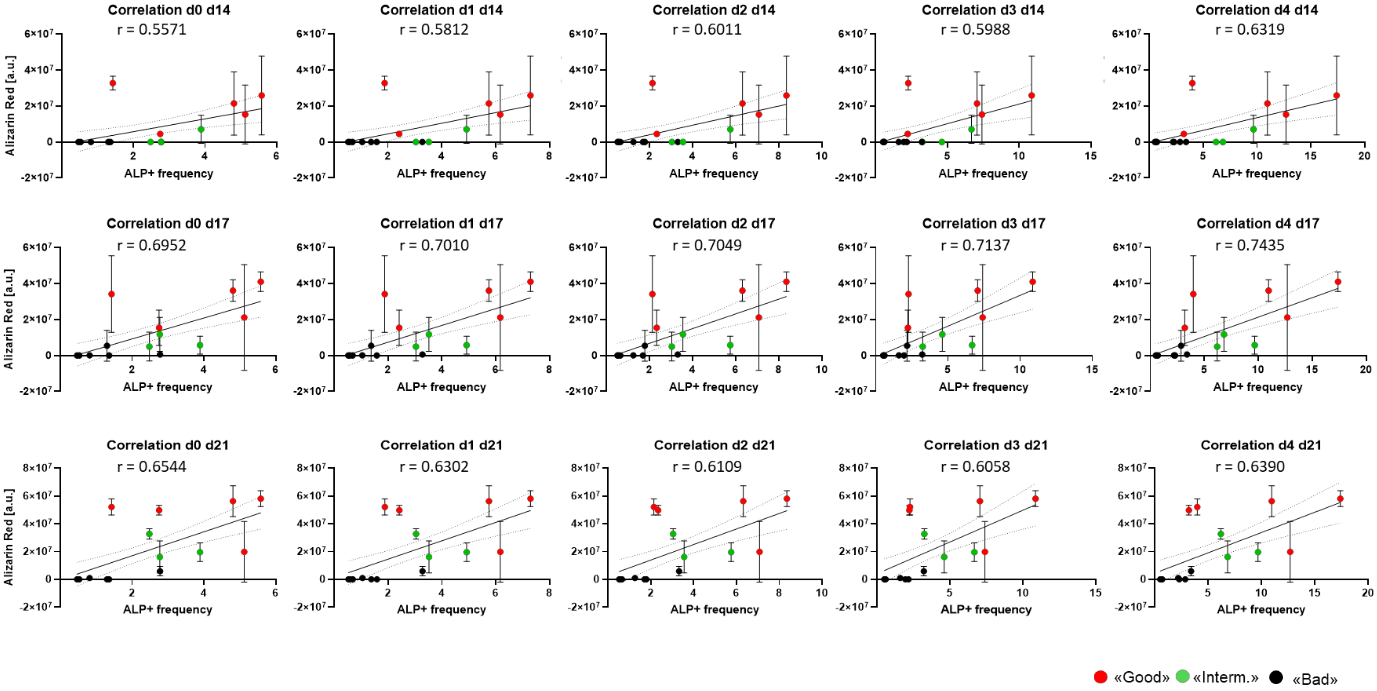

Correlation d0 d14
r = 0.5571
Correlation d1 d14
r = 0.5812
Correlation d2 d14
r = 0.6011
Correlation d3 d14
r = 0.5988
Correlation d4 d14
r = 0.6319
Correlation d0 d17
r = 0.6952
Correlation d1 d17
r = 0.7010
Correlation d2 d17
r = 0.7049
Correlation d3 d17
r = 0.7137
Correlation d4 d17
r = 0.7435
Correlation d0 d21
r = 0.6544
Correlation d1 d21
r = 0.6302
Correlation d2 d21
r = 0.6109
Correlation d3 d21
r = 0.6058
Correlation d4 d21
r = 0.6390
Alizarin Red [a.u.]
ALP+ frequency
«Good» «Interm.» «Bad»

A

Cells *Singlets*

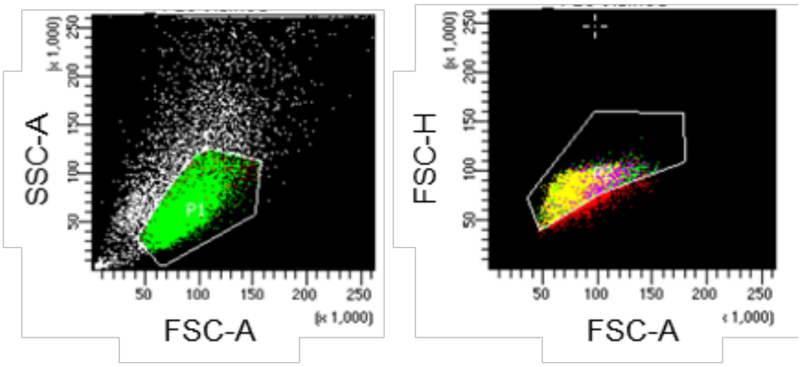

Stained

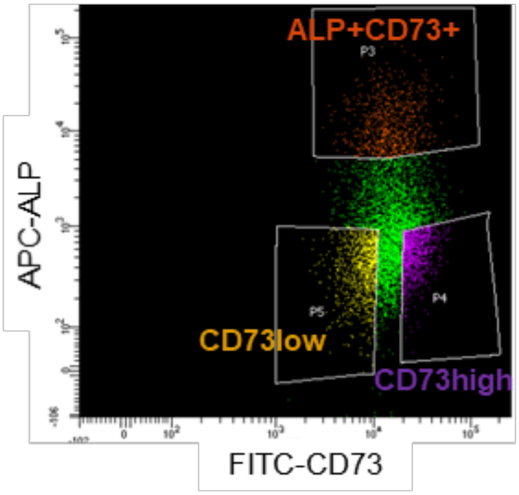

Tube: F28 stained

Population	#Events	%Parent	%Total
All Events	10,000	####	100.0
P1	7,119	71.2	71.2
P2	6,552	92.0	65.5
P3 ALP+CD73+	707	10.8	7.1
P4 CD73high	1,130	17.2	11.3
P5 CD73low	1,119	17.1	11.2

Figure S4

B

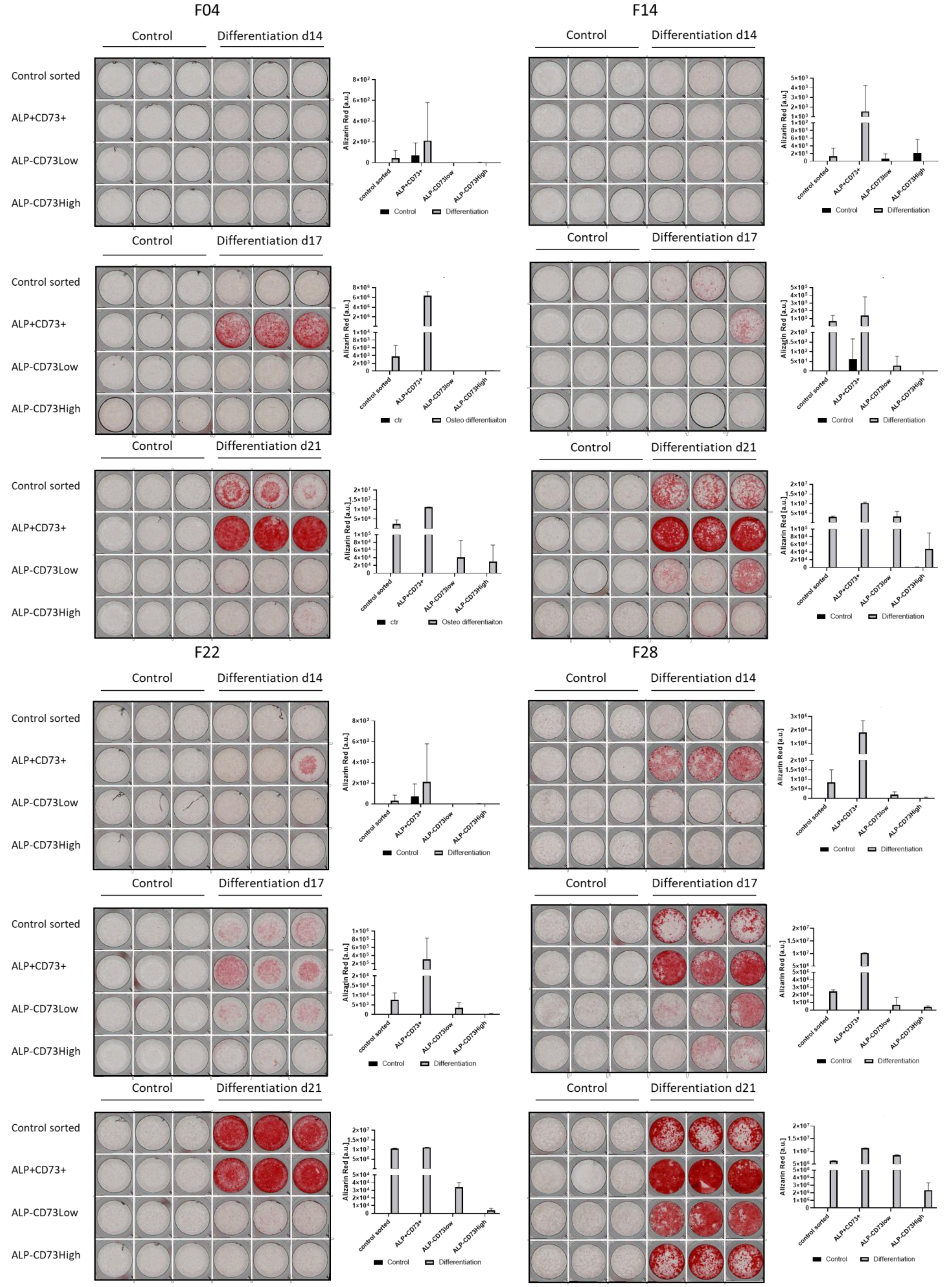

F04
Control
Differentiation d14
Control sorted
ALP+CD73+
ALP-CD73Low
ALP-CD73High
Alizarin Red [a.u.]
control sorted
ALP+CD73+
ALP-CD73low
ALP-CD73High
Control
Differentiation
Control
Differentiation d17
ctr
Osteo differentiaiton
Control
Differentiation d21
ctr
Osteo differentiaiton
F14
Control
Differentiation d14
Control
Differentiation d17
Control
Differentiation d21
F22
Control
Differentiation d14
Control
Differentiation d17
Control
Differentiation d21
F28
Control
Differentiation d14
Control
Differentiation d17
Control
Differentiation d21

Figure S4

C

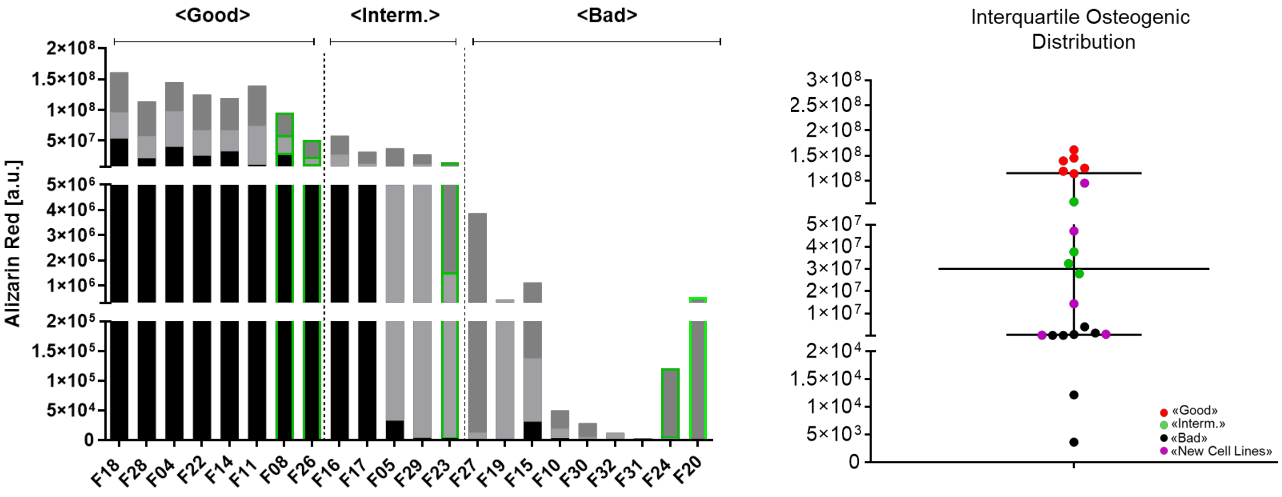

<Good>
<Interm.>
<Bad>
Alizarin Red [a.u.]
F18 F28 F04 F22 F14 F11 F08 F26 F16 F17 F05 F29 F23 F27 F19 F15 F10 F30 F32 F31 F24 F20
Interquartile Osteogenic Distribution
«Good»
«Interm.»
«Bad»
«New Cell Lines»

D

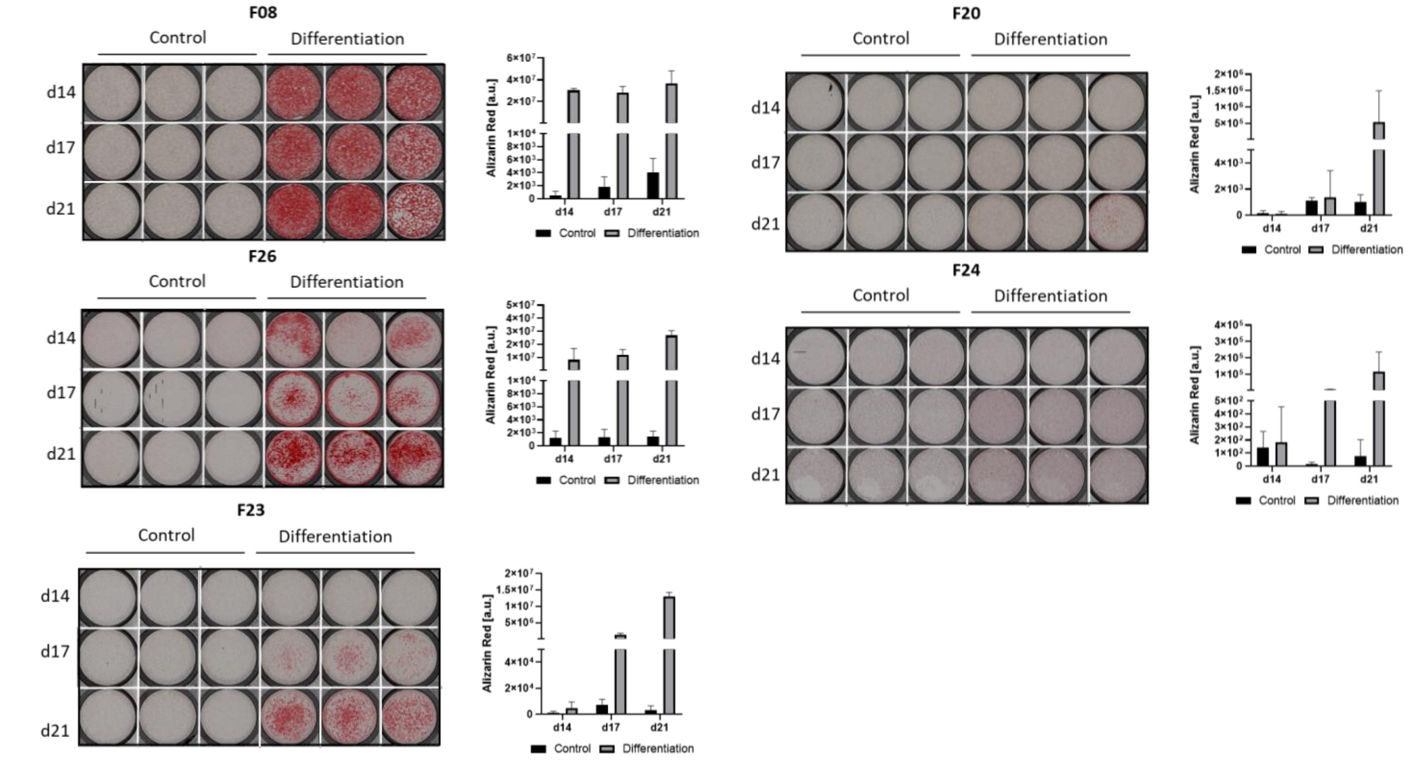

F08
Control
Differentiation
d14
d17
d21
F26
F23
F20
F24
Alizarin Red [a.u.]
Control
Differentiation

Figure S4

E

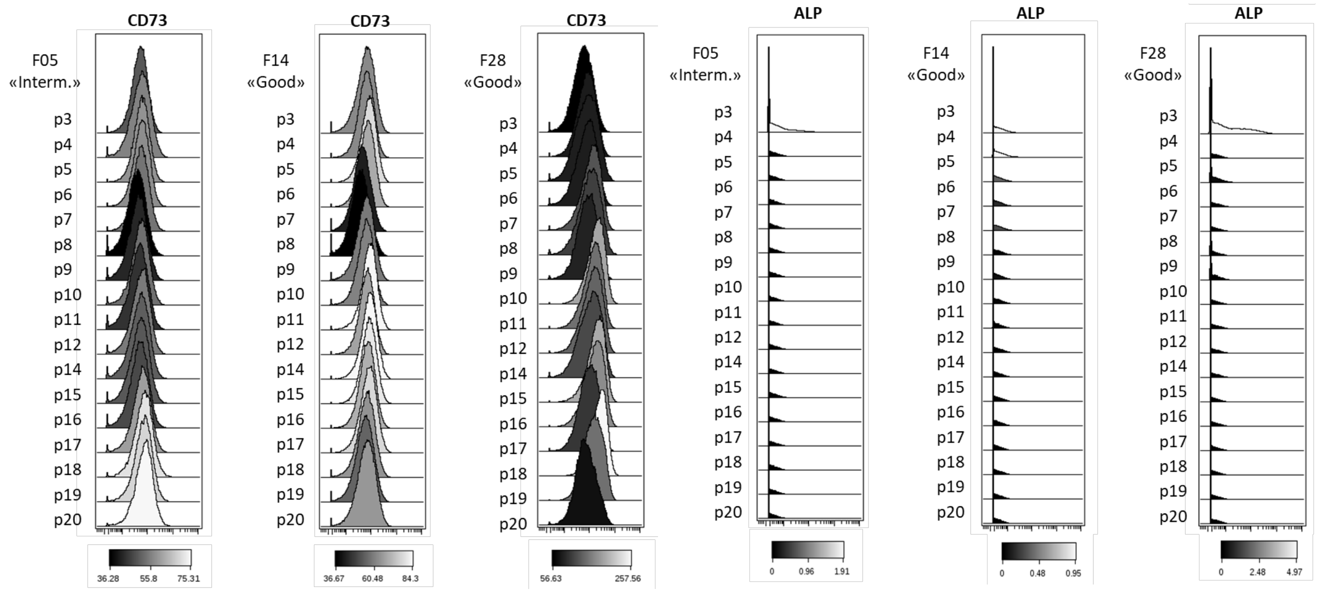
CD73
F05
«Interm.»
F14
«Good»
F28
«Good»
ALP
p3
p4
p5
p6
p7
p8
p9
p10
p11
p12
p14
p15
p16
p17
p18
p19
p20
36.28 55.8 75.31
36.67 60.48 84.3
56.63 257.56
0 0.96 1.91
0 0.48 0.95
0 2.48 4.97

F

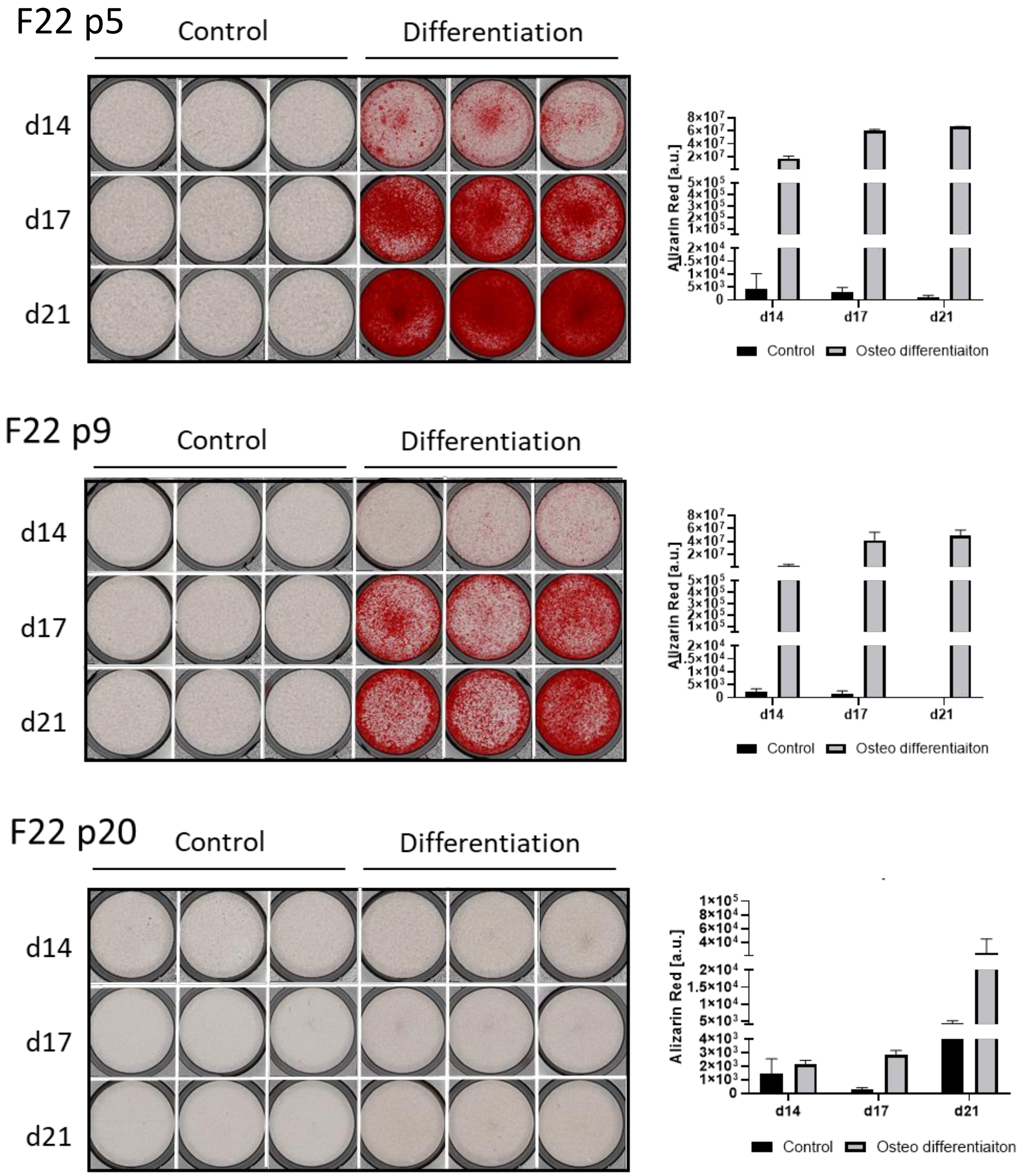
F22 p5
Control
Differentiation
d14
d17
d21
Alizarin Red [a.u.]
Control
Osteo differentiaiton
F22 p9
F22 p20

Figure S5

A

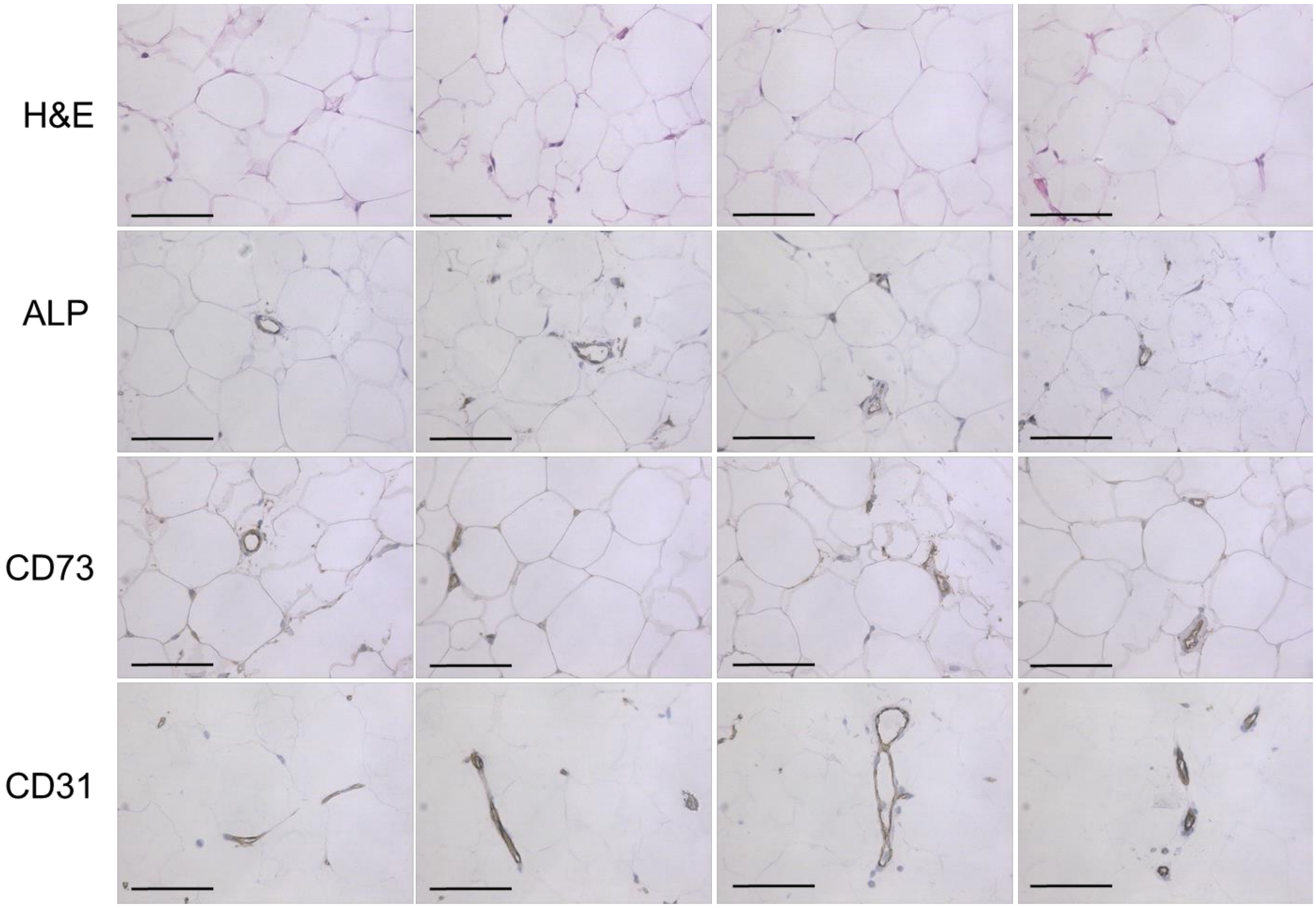
H&E
ALP
CD73
CD31

B

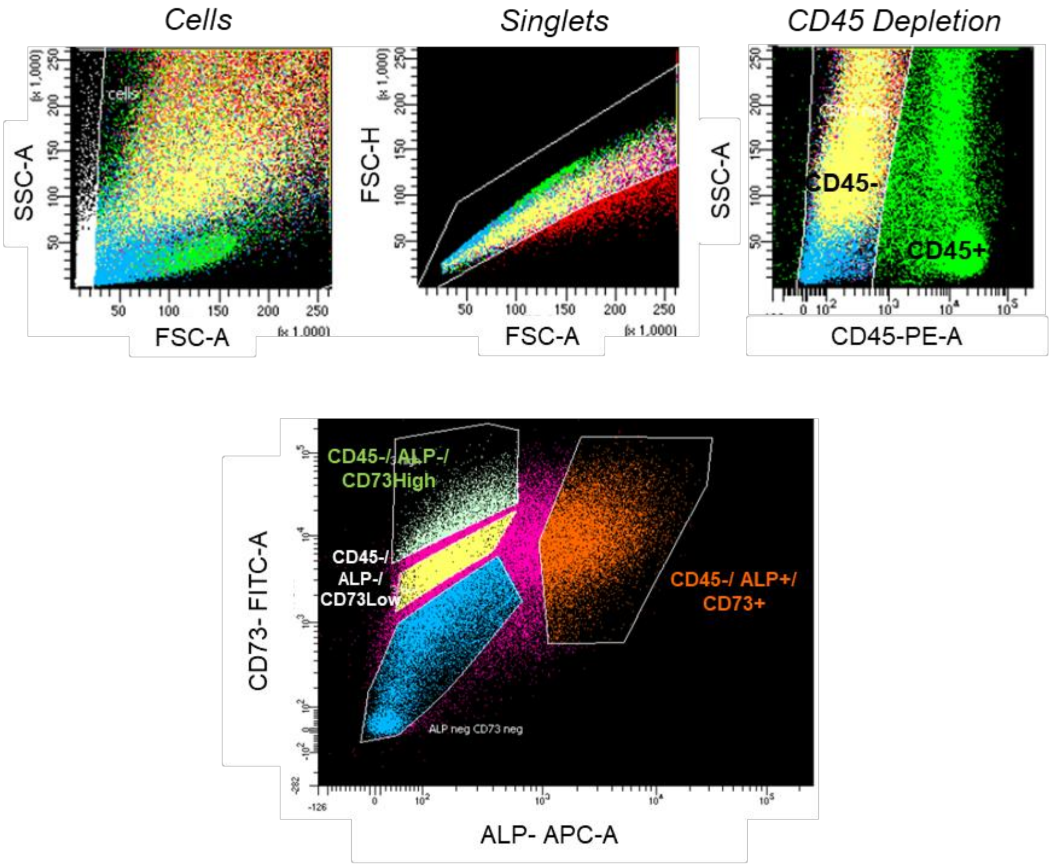
Cells
Singlets
CD45 Depletion
SSC-A
FSC-A
FSC-H
CD45-PE-A
cells
CD45-
CD45+
CD45-/ ALP-/ CD73High
CD45-/ ALP-/ CD73Low
CD45-/ ALP+/ CD73+
ALP neg CD73 neg
CD73- FITC-A
ALP- APC-A

Figure S5

C

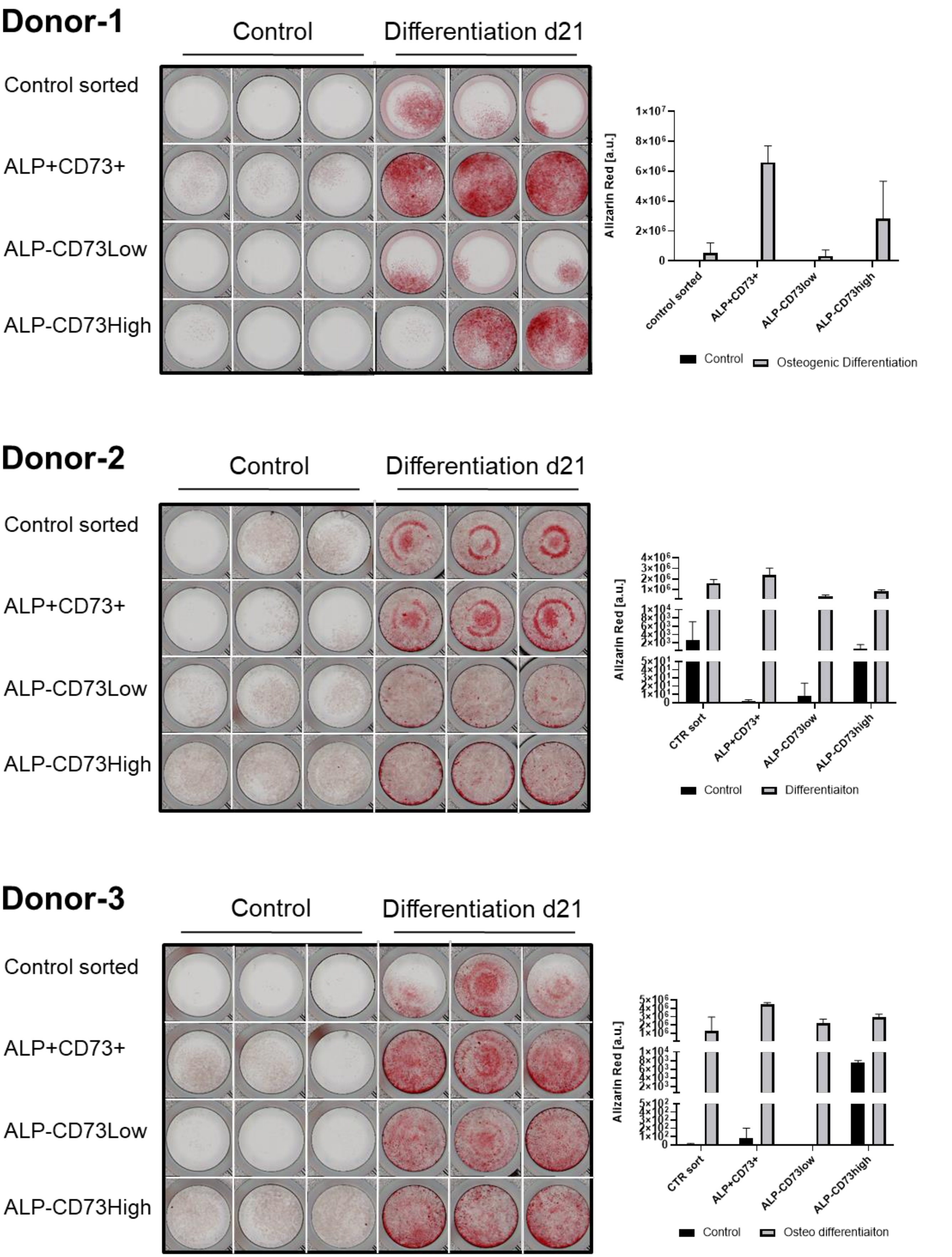

Donor-1
Control
Differentiation d21
Control sorted
ALP+CD73+
ALP-CD73Low
ALP-CD73High
Alizarin Red [a.u.]
control sorted
ALP+CD73+
ALP-CD73low
ALP-CD73high
Control
Osteogenic Differentiation
Donor-2
Control
Differentiation d21
Control sorted
ALP+CD73+
ALP-CD73Low
ALP-CD73High
Alizarin Red [a.u.]
CTR sort
ALP+CD73+
ALP-CD73low
ALP-CD73high
Control
Differentiaiton
Donor-3
Control
Differentiation d21
Control sorted
ALP+CD73+
ALP-CD73Low
ALP-CD73High
Alizarin Red [a.u.]
CTR sort
ALP+CD73+
ALP-CD73low
ALP-CD73high
Control
Osteo differentiaiton

Table S1. Mass cytometry antibody panel

Marker	AMU	Element isotope	Antibody clone	Staining concentration [μg/ml]
CD105	176	Yb	43A3	2.5
CD73	146	Nd	AD2	2.5
CD90	161	Dy	5.00E+10	2.5
CD14	175	Lu0	M5E2	0.625
CD11b	144	Nd	ICRF44	1.25
CD79a	156	Dy	HM47	1.25
HLA-DR	174	Yb	L243	2.5
CD19	142	Nd	HIB19	0.625
CD45	154	Sm	HI30	0.625
EGFR	167	Er	AY13	2.5
PDGFR	160	Gd	D13C6	2.5
CD44	166	Er	IM7	2.5
CD38	172	Yb	HIT2	10
Stro1	163	Dy	STRO-1	5
CD146	155	Gd	P1H12	0.625
CD106	158	Gd	STA	5
NG2	150	Sm	7.1	5
CD271	165	Ho	ME20.4	2.5
CD166	164	Dy	3A6	5
Ki67	147	Sm	1297A	1.25
ALP	169	Tm	TRA-2-49/6E	1.25
RUNX2	153	Eu	S533	1.25
MSX2	148	Nd	786607	5
Osterix/SP7	159	Tb	764704	5
LRP5	162	Dy	2B11	10
DLK1	149	Sm	211309	5
FABP4	171	Yb	2H3L2	5
PPARγ	168	Er	A3409A	5
Sox9	170	Er	3C10	1.25
CD325	143	Nd	8C11	10
AGCAN	151	Eu	179509	10

Table S2. Osteogenic differentiation barcoding schema

TUBE 1	cell line	day0	day1	day2	day3	day4	MIX1	MIX2
Good	F4	bc1	bc4	bc7	bc10	bc13	bc16	bc17
Bad	F19	bc2	bc5	bc8	bc11	bc14		
Good	F11	bc3	bc6	bc9	bc12	bc15		
**TUBE 2**	**cell line**	**day0**	**day1**	**day2**	**day3**	**day4**	**MIX1**	**MIX2**
Intermediate	F5	bc1	bc4	bc7	bc10	bc13	bc16	bc17
Bad	F27	bc2	bc5	bc8	bc11	bc14		
Good	F14	bc3	bc6	bc9	bc12	bc15		
**TUBE 3**	**cell line**	**day0**	**day1**	**day2**	**day3**	**day4**	**MIX1**	**MIX2**
Bad	F15	bc1	bc4	bc7	bc10	bc13	bc16	bc17
Bad	F10	bc2	bc5	bc8	bc11	bc14		
Intermediate	F17	bc3	bc6	bc9	bc12	bc15		
**TUBE 4**	**cell line**	**day0**	**day1**	**day2**	**day3**	**day4**	**MIX1**	**MIX2**
Good	F18	bc1	bc4	bc7	bc10	bc13	bc16	bc17
Bad	F30	bc2	bc5	bc8	bc11	bc14		
Good	F28	bc3	bc6	bc9	bc12	bc15		
**TUBE 5**	**cell line**	**day0**	**day1**	**day2**	**day3**	**day4**	**MIX1**	**MIX2**
Good	F22	bc1	bc4	bc7	bc10	bc13	bc16	bc17
Bad	F32	bc2	bc5	bc8	bc11	bc14		
Intermediate	F29	bc3	bc6	bc9	bc12	bc15		
**TUBE 6**	**cell line**	**day0**	**day1**	**day2**	**day3**	**day4**	**MIX1**	**MIX2**
Bad	F31	bc2	bc5	bc8	bc11	bc14	bc16	bc17
Intermediate	F16	bc3	bc6	bc9	bc12	bc15		

Barcode: bc

Table S3. Barcoding plan prediction experiment

New ASCs	F20	F8	F26	F21	F24	F23					
**Barcode**	bc 1	bc 2	bc 3	bc 4	bc 5	bc 6					
**Reference**	**F5**	**F22**	**F30**	**F32**	**F4**	**F14**	**F15**	**F31**	**F18**	**F28**	**MIX**
**Barcode**	bc 9	bc 10	bc 11	bc 12	bc 13	bc 14	bc 15	bc 16	bc 17	bc 18	bc 19

Barcode: bc

Table S4: Barcoding plan for the passage experiment

Tube1	passage	bc	Tube2	passage	bc	Tube3	passage	bc	Tube4	passage	bc
**F22**	**p3**	bc 1	**F28**	**p3**	bc 1	**F14**	**p3**	bc 1	**F5**	**p3**	bc 1
**F22**	**p4**	bc 2	**F28**	**p4**	bc 2	**F14**	**p4**	bc 2	**F5**	**p4**	bc 2
**F22**	**p5**	bc 3	**F28**	**p5**	bc 3	**F14**	**p5**	bc 3	**F5**	**p5**	bc 3
**F22**	**p6**	bc 4	**F28**	**p6**	bc 4	**F14**	**p6**	bc 4	**F5**	**p6**	bc 4
**F22**	**p7**	bc 5	**F28**	**p7**	bc 5	**F14**	**p7**	bc 5	**F5**	**p7**	bc 5
**F22**	**p8**	bc 6	**F28**	**p8**	bc 6	**F14**	**p8**	bc 6	**F5**	**p8**	bc 6
**F22**	**p9**	bc 7	**F28**	**n.d.**	-	**F14**	**p9**	bc 7	**F5**	**p9**	bc 7
**F22**	**p10**	bc 8	**F28**	**p10**	bc 7	**F14**	**p10**	bc8	**F5**	**p10**	bc 8
**F22**	**p11**	bc 9	**F28**	**p11**	bc 8	**F14**	**p11**	bc9	**F5**	**p11**	bc 9
**F22**	**p12**	bc 10	**F28**	**p12**	bc 9	**F14**	**n.d.**	-	**F5**	**n.d.**	-
**F22**	**n.d.**	-	**F28**	**p13**	bc 10	**F14**	**p13**	bc 10	**F5**	**p13**	bc 10
**F22**	**p14**	bc 11	**F28**	**p14**	bc 11	**F14**	**p14**	bc 11	**F5**	**p14**	bc 11
**F22**	**p15**	bc 12	**F28**	**p15**	bc 12	**F14**	**p15**	bc 12	**F5**	**p15**	bc 12
**F22**	**p16**	bc 13	**F28**	**p16**	bc 13	**F14**	**p16**	bc 13	**F5**	**p16**	bc 13
**F22**	**p17**	bc 14	**F28**	**p17**	bc 14	**F14**	**p17**	bc 14	**F5**	**p17**	bc 14
**F22**	**p18**	bc 15	**F28**	**p18**	bc 15	**F14**	**p18**	bc 15	**F5**	**p18**	bc 15
**F22**	**p19**	bc 16	**F28**	**p19**	bc 16	**F14**	**p19**	bc 16	**F5**	**p19**	bc 16
**F22**	**p20**	bc 17	**F28**	**p20**	bc 17	**F14**	**p20**	bc 17	**F5**	**p20**	bc 17
	**MIX1**	bc 18		**MIX1**	bc 18		**MIX1**	bc 18		**MIX1**	bc 18
	**MIX2**	bc 19		**MIX2**	bc 19		**MIX2**	bc 19		**MIX2**	bc 19

Not done: n.d.; Barcode: bc

Supplemental Titles and Legends

Figure S1. *In vitro* chondrogenic and adipogenic categorization of 17 AD-MSCs

A) Sum of the pixels acquired at the three time points (day 14, 17, 21) for chondrogenic (left) and adipogenic (right) differentiation of all 17 AD-MSC lines and interquartile categorization into «good», «intermediate», and «bad» AD-MSCs. **C)** Summary of the categorization of all 17 AD-MSCs for the three differentiation lineages (osteogenic, chondrogenic, and adipogenic). interm. = intermediate

Figure S2. UMAP analyses in the 17 human AD-MSC lines

A) UMAP projections of all 31 markers in 17 AD-MSC lines. Each dot represents one cell. Blue denotes minimal, green intermediate, and red high expression.

Figure S3. Analyses of the osteogenic subpopulation

A) Means of the percentage of alkaline phosphatase (ALP) positive cells and CD73 positive cells in the three AD-MSC categories during the five analyzed days of osteogenic differentiation (d0, d1, d2, d3, d4). Error bars represent the mean ± s.d. of the percentage of positive cells present in «good» (n = 6), «intermediate» (n = 4), and «bad» (n = 7) AD-MSCs. **B)** Pearson correlations of the ALP frequency measured by CyTOF at day 0, 1, 2, 3, 4 with the staining intensities measured at day 14, 17, and 21 for osteogenic differentiation. Red dots represent «good», green «intermediate» (interm.), and black «bad» differentiating lines. Error bars indicate the triplicates of the staining and are presented as mean ± s.d. For statistical analyses, the one-way ANOVA Dunnett's multiple comparisons test was used to compare each day of the "good" AD-MSCs with the same day of "intermediate" and "bad" categories: * $p \leq 0.05$, ** $p \leq 0.01$, *** $p \leq 0.001$, and **** $p \leq 0.0001$. ns=not significant.

Figure S4. ALP+/CD73+ Sorting analysis and prediction of osteogenic differentiation potential

A) Gating strategy for FACS sorting for the following subpopulations: ALP+/CD73+, ALP-/CD73low, and ALP-/CD73high. **B)** Alizarin Red staining and quantification of the sorted subpopulations in four AD- MSC lines (F04, F14, F22, F28) after 14, 17, and 21 days. Control sorted are unstained cells, which were run through the FACS sorting machine. Depicted are triplicates of undifferentiated cells (control) and cells cultured with the differentiation medium (differentiation). Error bars indicate the triplicates of the staining and are presented as mean ± s.d. **C)** Categorization of the new AD-MSC lines (depicted in green) together with all the 17 already analyzed lines, based on Alizarin Red quantification after 14, 17, and 21 days of osteogenic differentiation and interquartile distribution of the five new AD-MSCs (depicted in violet). **D)** Alizarin Red staining and quantification of five new AD-MSCs: two «good» (F08, F26), one «intermediate» (F23), and two «bad» (F20, F24). Depicted are triplicates of undifferentiated cells (control) and cells cultured under osteogenic differentiation conditions (differentiation). Error bars indicate triplicates of the staining and are presented as mean ± s.d. **E)** Histograms of median intensities of expression of selected markers (CD73 and ALP) in F05, F14, F22 and F28 AD-MSC lines from passage 3 (p3) till passage 20 (p20). Black is the lowest intensity and white represents the highest intensity. **F)** Alizarin Red staining and quantification of F22 at passage p5, p9, and p20 after 14, 17, and 21 days of osteogenic differentiation. Depicted are triplicates of undifferentiated cells (control) and cells

cultured under osteogenic differentiation medium (differentiation). Error bars indicates the triplicates of the staining and are presented as mean ± s.d.

Figure S5. ALP+/CD73+ cells are present in the human fat tissue and stromal vascular fraction

A) Hematoxylin/Eosin (H&E) and immunohistochemistry staining of human fat tissue for ALP, CD73, and CD31. Scale 100 µm. **B)** Gating strategy for sorting the selected subpopulations (CD45- /ALP+/CD73+, CD45- /ALP-/CD73low, CD45-/ALP-/CD73high) in the SVFs. **C)** Alizarin Red staining and pixel quantification of sorted SVF fractions (CD45-/ALP+/CD73+, CD45-/ALP-/CD73low, CD45-/ALP- /CD73high) after 21 days of osteogenic differentiation *in vitro*. Control sorted are unstained SVFs, which were run through the FACS sorting machine. Depicted are triplicates of undifferentiated cells (control) and cells cultured with osteogenic differentiation medium (differentiation). Error bars indicate the triplicates of the staining and are presented as mean ± s.d.
